# Hardware Implementation of On‐Chip Hebbian Learning Through Integrated Neuromorphic Architecture

**DOI:** 10.1002/adma.202506920

**Published:** 2025-06-25

**Authors:** Seonkwon Kim, Seongil Im, In Cheol Kwak, Jungwha Lee, Dong Gue Roe, Hyunsu Ju, Jeong Ho Cho

**Affiliations:** ^1^ Department of Chemical and Biomolecular Engineering Yonsei University Seoul 03722 Republic of Korea; ^2^ Center of Quantum Technology Korea Institute of Science and Technology (KIST) Seoul 02792 Republic of Korea

**Keywords:** artificial synapse, artificial neuron, neuromorphic computing, neuromorphic devices, on‐chip learning

## Abstract

The von Neumann bottleneck and growing energy demands of conventional computing systems require innovative architectural solutions. Although neuromorphic computing is a promising alternative, implementing efficient on‐chip learning mechanisms remains a fundamental challenge. Herein, a novel artificial neural platform is presented that integrates three synergistic components: modulation‐optimized presynaptic transistors, threshold switching memristor‐based neurons, and adaptive feedback synapses. The platform demonstrates real‐time synaptic weight modification through correlation‐based learning, effectively implementing Hebbian principles in hardware without requiring extensive peripheral circuitry. Stable device operation and successful implementation of local learning rules are confirmed by systematically characterizing a 6 × 6 array configuration. The experimental results demonstrate a correlation between input–output signals and subsequent weight modifications, establishing a viable pathway toward hardware implementation of Hebbian learning in neuromorphic systems.

## Graphical abstract

This work presents a neuromorphic hardware platform that integrates presynaptic transistors, threshold switching neurons, and adaptive feedback synapses. The architecture enables on‐chip Hebbian learning through correlation‐based weight updates without external training circuits. A 6 × 6 device array demonstrates stable real‐time operation, offering a pathway toward efficient, scalable brain‐inspired computing systems.

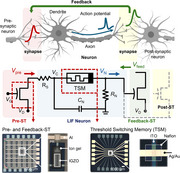

## 1 Introduction

Neuromorphic computing systems offer an alternative method to overcome the fundamental limitations of conventional complementary metal‐oxide semiconductor (CMOS) electronics and von Neumann architectures.^[^ ^1^, ^2^, ^3^ ^]^ As modern computing demands continue to increase, traditional architectures^[^ ^3^, ^4^ ^]^ encounter significant challenges in terms of energy consumption and processing efficiency, particularly due to the physical separation between memory and processing units.^[^ ^5^, ^6^ ^]^ Neuromorphic systems address these challenges by emulating the brain's architectural principles, providing substantial energy efficiency benefits through co‐located memory and computing elements, and enabling enormous parallel processing through distributed computing nodes.^[^ ^7^, ^8^, ^9^, ^10^, ^11^, ^12^ ^]^ In this domain, Hebbian learning mechanisms have emerged as an effective method for the hardware implementation of neural networks.^[^ ^13^, ^14^ ^]^ This learning principle provides a fundamental method for modifying connection strengths between computing elements, enabling the system to adapt based on the correlation between input and output signals The fundamental characteristic of Hebbian learning is its localized operation, where connection strength modifications depend solely on the activity of directly connected elements, eliminating the need for complex external control signals or sophisticated training algorithms. This localized learning property makes it particularly suitable for hardware implementation. However, the realization of Hebbian learning in practical hardware systems remains significantly challenging.

While the implementation of individual neural network components has progressed owing to recent advances in neuromorphic devices, the realization of learning and adaptation during operation remains a significant challenge.^[^ ^15^, ^16^, ^17^, ^18^ ^]^ Current approaches have two major limitations. First, most systems rely on off‐chip training, where the learning process occurs separately from the device operation.^[^ ^19^, ^20^, ^21^ ^]^ Second, they often require extensive CMOS circuitry to process signals and update connection strengths, thereby undermining the potential advantages of neuromorphic systems.^[^ ^22^, ^23^ ^]^ A critical technical challenge in implementing the basic principle of Hebbian learning in hardware is determining how to modify the connection strength (Δ*w*) based on the correlation between input (x) and output (y) signals. While prior implementations have made progress in realizing Hebbian‐like plasticity using memristive or transistor‐based devices, the learning signal (Δ*w*) is typically an implicit outcome of spike timing or pulse overlap, rather than a directly observable quantity. For instance, Hansen et al. demonstrated memristive crossbar arrays for Hebbian learning, yet required external systems to monitor and interpret weight updates from device behaviors.^[^ ^24^ ^]^ Such approaches still depend on off‐chip processing or digital feedback, limiting their ability to autonomously adapt during operation and increasing system complexity and energy consumption. Moreover, the additional circuitry required for these implementations increases system complexity and power consumption, undermining the original benefits of neuromorphic computing.

This manuscript presents an artificial neural platform (ANP) that offers a practical solution to these challenges by leveraging a carefully designed circuit architecture. Our system integrates three essential components that operate synergistically enable on‐chip learning: a pre‐synaptic transistor (pre‐ST) that precisely controls how input signals are transmitted, a threshold switching memristor (TSM)‐based leaky‐integrate‐and‐fire (LIF) neuron that generates output signals when sufficient input is received, and a feedback synaptic transistor (feedback‐ST) that modifies connection strengths based on the correlation between input and output signals. This architecture is specifically designed to minimize reliance on external CMOS circuits while maintaining the ability to process multiple input signals simultaneously. By implementing these components in a 6 × 6 array configuration, we demonstrate the feasibility of achieving on‐chip learning capabilities with minimal external circuitry. Our experimental results indicate that connection strengths can be modified during operation based on the correlation between input and output signals, indicating potential for hardware‐based learning systems. This work provides insights into how Hebbian learning principles can be effectively implemented in hardware, and it establishes a framework for developing more efficient neuromorphic computing systems. The results suggest the possibility of creating adaptive systems that can learn from incoming information during operation while maintaining the efficiency advantages of neuromorphic architectures.

## 2 Result and Discussion

### 2.1 Implementation of Artificial Synapse–Neuron Integration

**Figure 1:**
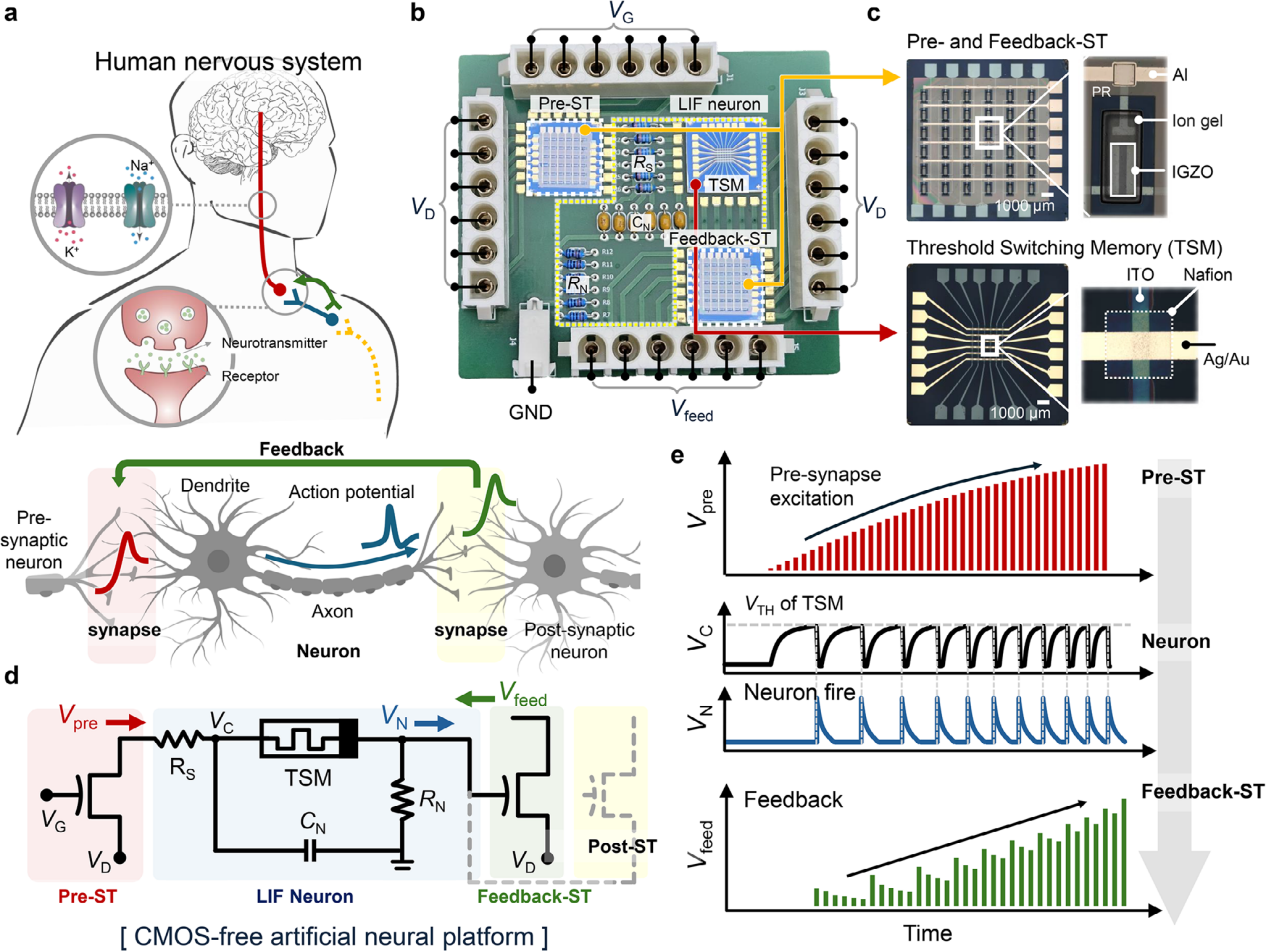
System architecture of the ANP. a) Schematic illustration of biological neural signal transmission displaying neurotransmitter release and action potential propagation. b) Hardware implementation of the ANP consisting of three key components: a pre‐ST, a TSM‐based neuron, and a feedback‐ST integrated on a PCB. c) Optical micrograph of the fabricated 6 × 6 array integrating STs and TSM neurons on a PCB. d,e) Circuit diagram and signal transmission pathway showing the operation principle of the ANP: input signals (*V*_D_) are modulated by *V*_G_, generating a pre‐synaptic output (*V*_pre_) that feeds into the neuron circuit for integration and firing of the neuron output (*V*_N_), which modulates *V*_D_ and ultimately produces a feedback signal (*V*_feed_) through the feedback‐ST.

**Figure** **1**a provides a schematic illustration of the human nervous system, highlighting the neural signal propagation and synaptic transmission mechanisms. The signal transmission in biological neurons involves a cascade of events: An incoming action potential triggers the release of neurotransmitters from the presynaptic terminal into the synaptic cleft. These neurotransmitters bind to receptors on the neuron's dendrites, inducing local membrane potential changes. When this potential exceeds a threshold at the soma, it triggers an action potential there. This action potential subsequently propagates along the axon, ultimately initiating neurotransmitter release at subsequent synaptic terminals. This biological architecture inspired the design of our ANP, which captures the essential features of neural information processing. The ANP comprises three primary components (Figure 1b): a pre‐ST for input signal modulation, a TSM‐based LIF neuron for threshold‐dependent signal generation, and a feedback‐ST for the dynamic adjustment of synaptic weights based on input–output correlations.

Three device arrays were integrated onto a printed circuit board (PCB) (Figure 1b,c): a 6 × 6 array of ion gel‐based synaptic transistors (STs) functioning as pre‐synaptic STs, another 6 × 6 array of ion gel‐based STs serving as feedback‐ST, and a 6 × 6 array of Nafion‐based TSMs. Peripheral electrical components, such as resistors and capacitors, were also included in the PCB to implement an artificial neuron circuit. The ST array was fabricated by sputtering indium tin oxide (ITO) source and drain electrodes onto an SiO_2_/Si substrate, followed by depositing an indium–gallium–zinc‐oxide (IGZO) active channel. A low‐k SU‐8 photoresist was spin‐coated and patterned using conventional photolithography to form an isolation layer and a via‐hole for further fabrication. An Al gate electrode was then deposited, and an ion gel containing 1‐ethyl‐3‐methylimidazolium bis(trifluoromethylsulfonyl)imide ([EMIM]^+^[TFSI]^−^) ionic liquids was deposited and patterned. For the LIF neuron array, the TSM array was fabricated by first sputtering an ITO bottom electrode onto a SiO_2_/Si substrate, followed by spin‐coating a Nafion film. The Nafion film, which contained an azide‐based photo‐cross‐linker was photopatterned (as detailed in our previous publication),^[^ ^25^ ^]^ and an Ag/Au top electrode was thermally evaporated.

Figure 1d,e presents the schematic circuit diagram and signal transmission process of the ANP, which includes a pre‐ST, a TSM‐based LIF neuron circuit, and a feedback‐ST. During operation, a consistent pulse signal (*V*_D_) is applied to the drain terminals of the STs, while the gate voltage (*V*_G_) modulates their conductance, gradually potentiating the output signal of the pre‐STs (*V*_pre_). This presynaptic output is then connected to the neuron circuit, where a capacitor (*C*_N_) integrates the incoming signal. When the potential across the capacitor exceeds the threshold voltage (*V*_TH_) of the TSM, the TSM switches from a high‐resistance state (HRS) to a low‐resistance state (LRS), rapidly discharging the capacitor and producing the neuron output (*V*_N_). This mechanism effectively mimics the action potential generation observed in biological neurons. The *V*_N_ is subsequently connected to the gate terminal of the feedback‐ST, where it modulates its conductance, resulting in a gradual potentiation of the feedback signal (*V*_feed_). The term “feedback” reflects the role of this component in implementing Hebbian learning, which will be discussed in detail later. A key advantage of our design is the elimination of additional CMOS‐based peripheral circuits, such as operational amplifiers for signal amplification or polarity adjustment. This simplification was achieved by utilizing three‐terminal STs, where the external drain voltage at each layer negates the need for such amplification circuits. By optimizing the material engineering and device architecture, we successfully eliminated the requirement for supplementary circuitry, enabling efficient system integration. Theoretically, this ABA configuration (where A represents the ST, and B represents the neuron circuit) can be extended infinitely. Specifically, by connecting the *V*_N_ to the drain terminal of another synapse (highlighted in yellow in Figure 1d), it can serve as a presynaptic input for subsequent neural units. This architectural flexibility enables the development of more complex neural networks while maintaining the efficient design principles of the system, significantly expanding the potential applications of our ANP platform.

### 2.2 Performances and Signal Modulation of the STs

**Figure 2:**
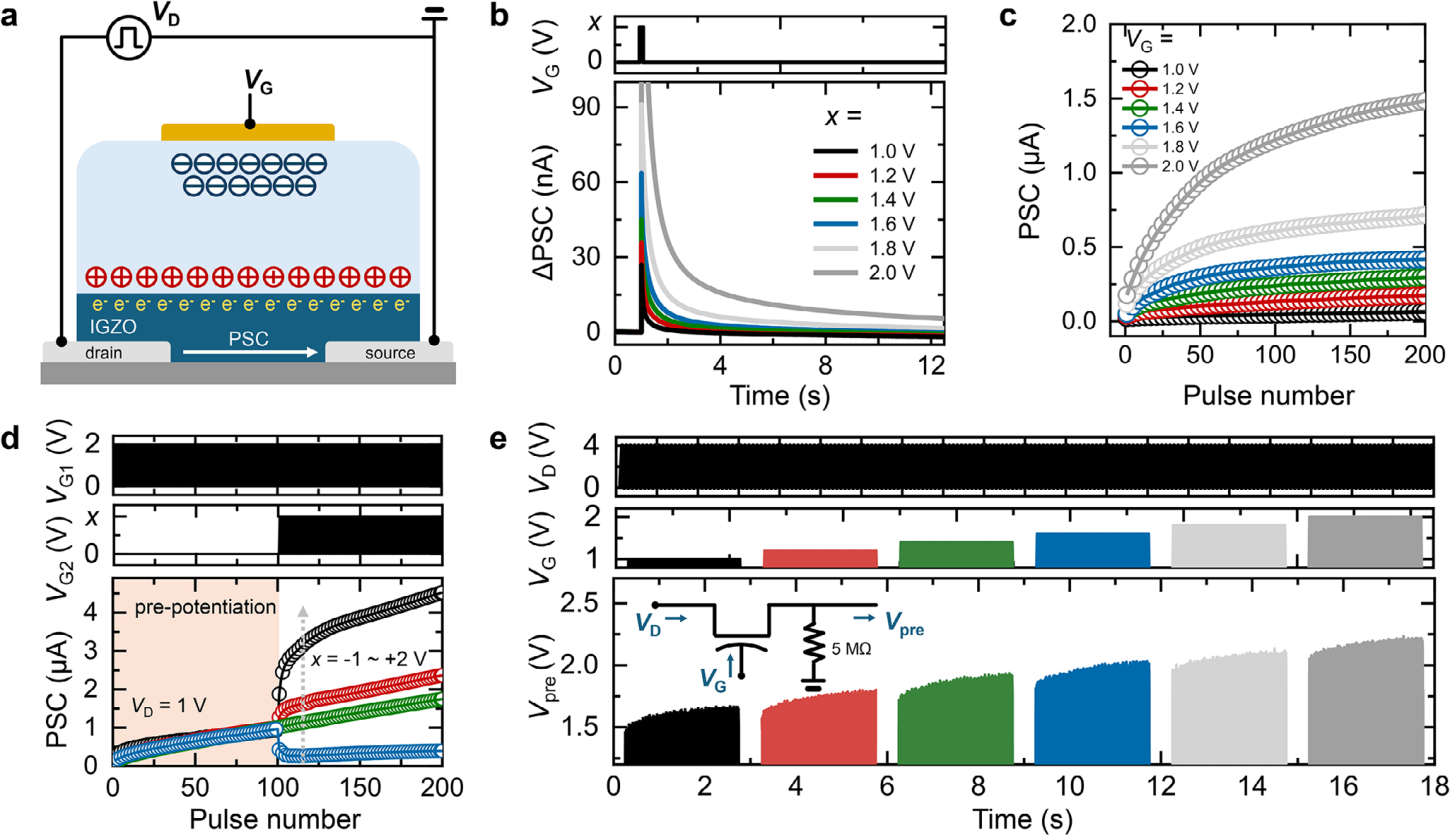
Characterization of the STs. a) Schematic of the ion gel‐gated ST employing IGZO as the active channel. b) EPSC characteristics demonstrating short‐term plasticity. c) LTP behavior demonstrating conductance modulation under repeated pulses. d) Multigate operation showing signal integration under fixed *V*_G1_ (+2 V, 50 Hz) and varying *V*_G2_ (−1 to +2 V). e) Presynaptic output voltage (*V*_pre_) modulation under simultaneous drain and gate pulse inputs, demonstrating voltage control capabilities.

Precise signal modulation of the STs is critical for implementing the ANP. **Figure** **2**a shows a schematic of the ion gel‐gated single‐gate ST employing IGZO as the active channel, which was selected for its suitability as an *n*‐type transistor. This aligns with the concept that a positive neuron output signal is required at the gate terminal to effectively potentiate the ST. Moreover, despite their short‐term characteristics, IGZO‐based electric double‐layer (EDL) transistors were chosen over organic electrochemical transistors (OECTs) because EDL transistors demonstrate stable device performance, whereas OECTs suffer from electrochemical electrode coupling. This coupling has severe implications for both device and circuit performance, causing a threshold voltage roll‐off.^[^ ^26^ ^]^ Additionally, the ANP required a careful threshold voltage adjustment of the ST, which was achieved by modulating the gallium (Ga) ratio within the IGZO precursor (Figure S1, Supporting Information). This threshold voltage tuning is essential for operation in enhancement mode because it ensures that the device remains normally off‐state until input signals, such as neuron output signals, are applied to the gate. This design reduces power consumption and mirrors the operational principles of biological neural systems, thereby enhancing the biomimetic capabilities of the ANP. Figure S1b (Supporting Information) summarizes the threshold voltages of IGZO‐based STs with different Ga ratios, with a 48% Ga ratio selected as the optimal ratio for this research.

The synaptic performance of the IGZO‐based STs was thoroughly investigated, as shown in Figure 2b (excitatory postsynaptic current [EPSC]), Figure 2c (long‐term potentiation [LTP], Figure S2 (Supporting Information) for LTP at low‐amplitude and low‐frequency input conditions, and Figure S3 (Supporting Information, paired‐pulse facilitation [PPF]). To validate the uniformity of synaptic characteristics across the array, we examined the LTP response of all devices in the 6 × 6 array configuration (Figures S4 and S5, Supporting Information). Consistent with previous findings, the STs demonstrated enhanced EPSC and LTP characteristics as the input *V*_G_ increased, as summarized in Figure S6 (Supporting Information). The modulation of postsynaptic current (PSC) corresponds to precise *V*_pre_ control, which can be achieved by adjusting the *V*_D_ voltage drop within the device. Additionally, the synaptic response of the STs exhibited strong dependence on input pulse frequency and width (Figure S7, Supporting Information). To confirm the reliability of the STs, we further evaluated their electrical stability under bias stress (Figure S8, Supporting Information) and repeated long‐term potentiation/depression (LTP/D) cycling (Figure S9, Supporting Information), both showing consistent and stable operation. A significant advantage of neuromorphic devices is their ability to integrate and process analog signals at the device level, enabling efficient computation. This is crucial for the versatility of ANPs, as it allows them to combine multiple neuron output signals through a single synapse, similar to biological systems. Figure S10 (Supporting Information) shows a photograph of the dual‐gate ST, and the pulse response characteristics of the dual‐gate ST under various gate biasing conditions. Applying a positive *V*_G1_ at gate 1 allows the potentiation degree to be enhanced or reduced by applying additional voltage (*V*_G2_) to gate 2. Figure 2d demonstrates the signal integration characteristics of an ST under a fixed input voltage at gate terminal 1 (*V*_G1_ = +2 V, 50 Hz) and varying input voltages at gate terminal 2 (*V*_G2_ = −1, 0, +1, and +2 V) after 100 potentiation pulses were pre‐applied. At *V*_G2_ = −1 V, potentiation was inhibited, but as *V*_G2_ increased, potentiation was enhanced (see Figure S11, Supporting Information) for the individual and simultaneous gate operation characteristics of the dual‐gate STs).

Finally, to simulate neuronal circuit behavior, pulse inputs were applied simultaneously to the drain (*V*_D_) and gate (*V*_G_) terminals to observe the voltage drop when integrating the ST into the ANP (Figure 2e). Constant pulses (*V*_D_ = 4 V, 50 Hz) were applied to the drain terminal, while *V*_G_s ranging from 1 to 2 V were applied for potentiation, with the *V*_pre_ measured (see Figure S12, Supporting Information, for results obtained without inter‐pulse intervals between different *V*_G_ amplitudes). *V*_pre_ modulation was observed by either varying the potentiation time at a constant pulse amplitude or adjusting the pulse amplitude of *V*_G_, providing a solid foundation for integrating STs into ANPs in this research.

### 2.3 Implementation of the TSM‐Based Neuron Circuit

**Figure 3:**
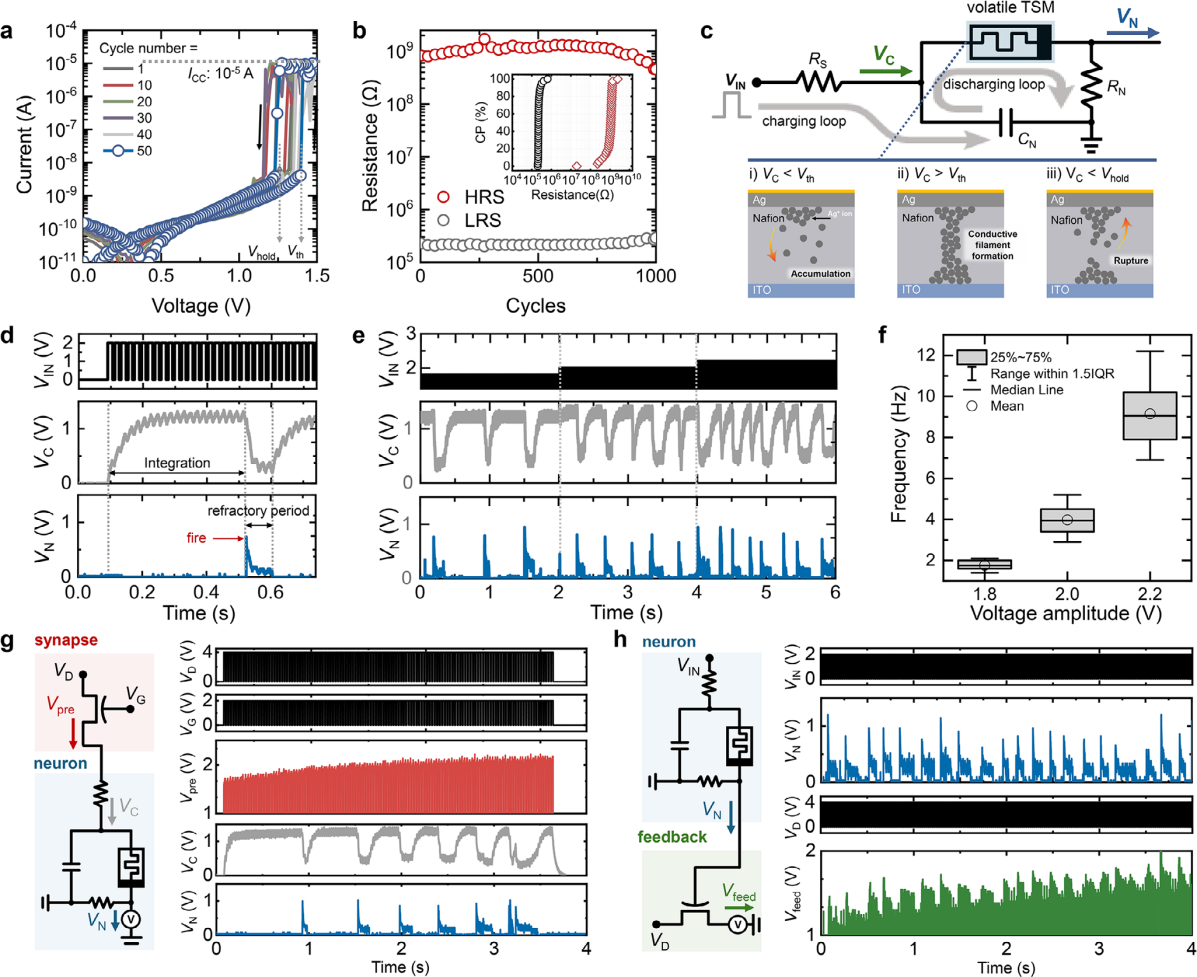
Implementation and characterization of the TSM‐based neuron circuit. a) Consecutive *I*–*V* curves displaying consistent threshold‐switching behavior under a 10 µA compliance current. b) Endurance characteristics over 1000 cycles with cumulative probability distributions of the HRS and LRS. c) Implementation diagram of the LIF neuron circuit showing RC integrator configuration and internal Ag ion dynamics at different stages. When *V*_C_ <*V*_TH_, Ag ions remain dispersed, maintaining the HRS. When *V*_C_ reaches *V*_TH_, the Ag ions form conductive filaments, transitioning the system to the LRS. When *V*_C_ drops below *V*_hold_, the Ag filaments rupture, returning the system to the HRS. d) Voltage characteristics demonstrating capacitor charging, threshold‐based firing, and discharge processes. e) Input–output relationship illustrating firing frequency modulation with varying input amplitudes. f) Statistical analysis of the firing frequency characteristics of 10 devices. g) Signal validation in the AB configuration demonstrating pre‐ST and neuron circuit interaction. h) BA configuration demonstrating neuron output integration with the feedback‐ST.

**Figure** **3**a shows the 50 consecutive current–voltage (*I*–*V*) curves of the TSM under a compliance current of 10 µA, with the voltage swept from 0 to 1.5 V and back to 0 V at the Ag electrode. The switching mechanism of our TSM device originates from Nafion's ability to host metallic filament formation because its microphase‐separated structure, which contains hydrophilic sulfonic acid groups, creates pathways for Ag ion migration.^[^ ^27^, ^28^ ^]^ The device switches from an HRS to an LRS at the *V*_TH_ of the TSM and reverts to the HRS when the voltage drops below the hold voltage (*V*_hold_). Both *V*_TH_ and *V*_hold_ remained consistent throughout the 50‐cycle test. The TSM demonstrated robust endurance over 1000 cycles, and the cumulative probability distributions of the HRS (measured at 0.1 V) and LRS (measured at 1.5 V) are shown in the inset of Figure 3b. To further evaluate device‐to‐device variability and structural effects on switching behavior, we investigated the switching behavior of all 36 TSM in the 6 × 6 array, and the impact of Nafion thickness (Figures S13,S14, and S15, Supporting Information).

The implementation and operating mechanisms of the LIF neuron circuit are illustrated in Figure 3c, with the corresponding voltage characteristics presented in Figure 3d. The LIF neuron circuit consists of an RC integrator where input voltage pulses (*V*_IN_ = +2 V, 50 Hz) charge the capacitor with a time constant *τ* = *R*_S·_ *C*_N_ (*R*_S_ = 470 kΩ; *C*_N_ = 100 nF). During operation, the voltage across the capacitor (*V*_C_) gradually increases through signal integration until it reaches *V*_TH_, whereupon the TSM suddenly switches from the HRS to LRS owing to the formation of an Ag conductive filament. This switch triggers the capacitor to rapidly discharge, generating an output pulse (*V*_N_). Subsequently, as *V*_C_ falls below *V*_hold_, the TSM returns to the HRS due to Rayleigh instability or Gibbs effect‐induced surface diffusion of metal atoms, initiating the next integration cycle.^[^ ^29^, ^30^ ^]^ To characterize the input–output relationship of the neuron circuit, we investigated the effect of varying input pulse amplitudes on the firing frequency (Figure 3e). As *V*_IN_ increased from 1.8 to 2.2 V, the frequency of *V*_N_ increased from 1.76 to 9.12 Hz. The statistical data obtained from 10 samples are summarized in Figure 3f. Further characterization of circuit parameters, including the effects of varying capacitance (*C*_N_) and series resistance (*R*_N_) in the discharging loop, are presented in Figure S16 (Supporting Information).

We connected the STs and neuron circuits to experimentally validate the signal propagation within the system (Figure 3g,h). Prior to establishing the complete ABA configuration (where A represents the synapse, and B represents the neuron), we examined the signals in both AB and BA configurations. In the AB configuration (Figure 3g), the pre‐ST was connected to the neuron circuit. Constant voltage pulses (*V*_D_ = 4 V, 50 Hz) were applied to the ST drain terminal, while constant potentiation pulses (*V*_G_ = 2 V, 50 Hz) were applied to the gate terminal. The ST output (*V*_pre_), which served as the input to the neuron circuit, was measured along with *V*_C_ and *V*_N_. Due to the potentiation pulses of *V*_G_, *V*_pre_ increased from 1.56 to 2.22 V, resulting in a gradual increase in the frequency of *V*_N_. Subsequently, we explored the BA configuration, where the neuron circuit output was connected to the gate terminal of the feedback‐ST (Figure 3h). Constant voltage pulses (*V*_IN_ = 2 V, 50 Hz) were applied to the input terminal of the neuron circuit while *V*_N_ and *V*_feed_ were measured. Considering that the input voltage pulses were constant, *V*_N_ maintained a steady firing frequency. These *V*_N_ pulses were connected to the gate terminal of the feedback‐ST, potentiating it and leading to an increase in *V*_feed_ from 1.12 to 1.6 V.

### 2.4 Hardware Implementation and Verification of On‐Chip Hebbian Learning

**Figure 4:**
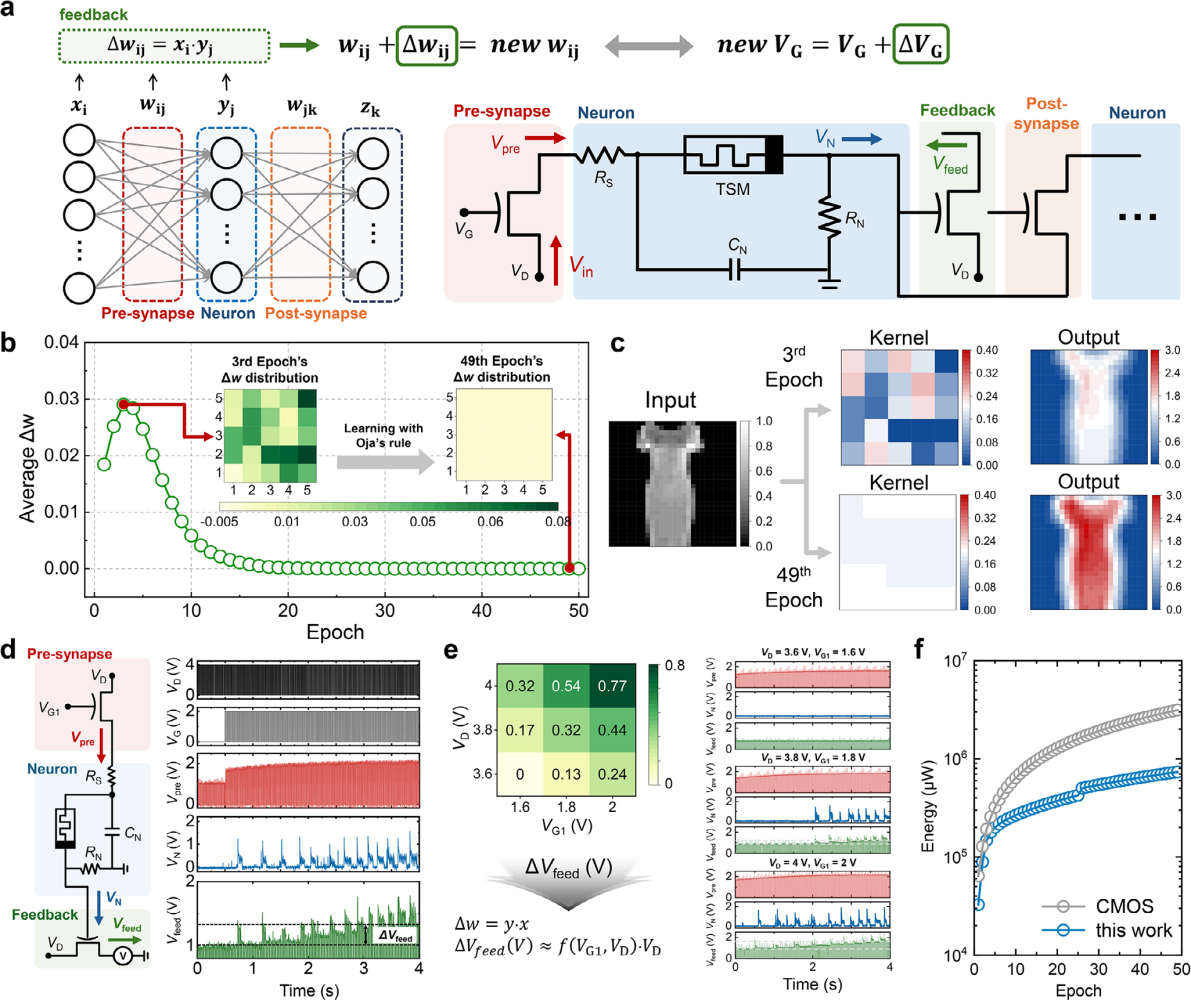
Hardware implementation and verification of Hebbian learning. a) System architecture illustrating the correspondence between the neural network components and physical circuit elements. b) Temporal evolution of the learning process showing weight modifications across training epochs. c) Visualization of kernel pattern evolution from initial random distribution to structured features. d) Temporal analysis of the circuit components exhibiting input–output signal propagation. e) Δ*V*_feed_ characterization with respect to *V*_G1_ and *V*_D_, demonstrating the implementation of the Hebbian weight update rule through circuit response. f) Total power consumption comparison between our circuit and CMOS implementation over 49 training epochs.

The systematic implementation of Hebbian learning in hardware requires precise correspondence between mathematical principles and physical circuit elements.^[^ ^31^ ^]^ **Figure** **4**a illustrates our system architecture, where a direct correlation is established between neural network components and circuit elements to realize on‐chip learning capability. The presynaptic input signal (*x*) in the neural network is implemented through the drain voltage (*V*_D_) in our circuit, while the synaptic weight (*w*) corresponds to the gate voltage (*V*_G1_) of the pre‐ST. When this presynaptic signal propagates through the circuit, it generates an output voltage (*V*_N_) from the neuron circuit. This neuronal output *V*_N_ corresponds to the neural network's output signal (*y*), and it functions as both an input to the postsynaptic circuit and a control signal for the feedback‐ST. As shown in Figure 4a, *V*_N_ serves as the gate voltage in the feedback‐ST. This configuration follows Ohm's Law to implement the product of *V*_N_ and *V*_D_ through *V*_feed_, which represents the correlation term (*x*·*y*) fundamental to Hebbian learning. The temporal change in this feedback voltage (Δ*V*_feed_) corresponds to the weight update term (Δ*w*) in Hebbian learning. This implementation enables direct hardware‐based learning without requiring external computation or additional control circuits.

To evaluate the proposed hardware implementation, we employed Oja's rule,^[^ ^32^ ^]^ a modified version of Hebbian learning, in neural network simulations. The incorporation of Oja's rule addresses the voltage stability requirements of our transistor‐based implementation by introducing a normalization term that constrains weight updates within the operational voltage range of the pre‐ST. Figure 4b demonstrates the temporal evolution of the unsupervised learning process. During the analysis at the third epoch, substantial modifications in specific weight kernels were observed, with weight changes (Δ*w*) reaching magnitudes of 0.03. These initial large updates indicate the active learning phase, where the system rapidly adjusts its synaptic weights. Subsequent analysis at the 49th epoch revealed significantly reduced weight adjustments (Δ*w* <0.005), indicating convergence to stable patterns. The learning progression exhibited a systematic reduction in update magnitudes, following the exponential decay typical of gradient‐based optimization. Figure 4c illustrates this convergence by depicting the evolution of kernel patterns, where the initial random weight distribution evolves into structured features without requiring ground‐truth labels. The functional efficacy was evaluated using Fashion MNIST dress images. The output patterns were found to demonstrate enhanced contrast ratios and feature definition after learning convergence, as observed in the 49th epoch, with the output signal amplitudes increasing by factors of 2–3 in relevant feature regions. Additionally, to provide quantitative performance validation, we implemented a standard two‐stage training protocol where our Hebbian‐pretrained network achieved 91.6% validation accuracy on Fashion‐MNIST, marginally exceeding conventional backpropagation (91.2%) with CNN model while eliminating label requirements during the computationally intensive phase (Table S1, Supporting Information).

Our implementation was experimentally validated through electrical characterization and array‐level integration. As shown in Figure 1b, we successfully fabricated a 6 × 6 array of our system that enables selective input application (Figures S17 and S18, Supporting Information). Figure 4d presents the temporal analysis results of individual circuit components within this architecture, demonstrating the detailed signal propagation pathway. The input signals consist of drain voltage (*V*_D_) and gate voltage (*V*_G1_), which interact in the presynaptic circuit to generate *V*_pre_. This presynaptic output signal is subsequently sent into the neuron circuit, where signal integration and a threshold‐based firing mechanism occur. The firing frequency increases proportionally with input signal strength, demonstrating the analog‐to‐temporal conversion capability of our circuit. These neuronal spikes, represented by *V*_N_, serve as a control signal for the feedback‐ST. The feedback‐ST processes these signals to generate *V*_feed_, and the temporal integration of this feedback signal produces the Δ*V*_feed_. This Δ*V*_feed_ determines how the synaptic weights, implemented through *V*_G1_ modifications, evolve during the learning process. Additionally, we demonstrated that the ANP can integrate multiple neural signals by combining two neuron outputs at a single feedback‐ST (Figure S19, Supporting Information), highlighting its potential for complex neuromorphic applications. Figure 4e illustrates how the output signal Δ*V*_feed_ of the circuit satisfies the Hebbian weight update rule Δ*w*. The heatmap characterizes Δ*V*_feed_ variations with respect to *V*_G1_ and *V*_D_, where *V*_N_ (corresponding to *y*) is expressed as a function of both *V*_G1_ and *V*_D_ (*y* = *f*(*V*_G1_,*V*_D_)). To modulate *V*_N_, we controlled *V*_G1_ and *V*_D_ to achieve different output levels. The heatmap demonstrates that Δ*V*_feed_, expressed as *f*(*V*_G1_,*V*_D_) ·*V*_D_, effectively implements the Hebbian learning rule of *y*·*x*. To verify this behavior, we measured the temporal evolution of circuit signals (*V*_pre_, *V*_N_, and *V*_feed_) at specific combinations of *V*_G1_ and *V*_D_, as shown in the bottom panels. Since the core operation of Hebbian learning is the multiplication of input and output signals (*x·y*), and both x and y are analog signals, we compare our circuit's energy efficiency to a conventional CMOS analog multiplier. Each Δ*w* update in a single transistor within the feedback‐ST consumes energy in the range of 1 to 3.24 µW, whereas a conventional CMOS analog multiplier requires 32 µW per operation. Figure 4f presents the total power consumption comparison across 49 training epochs. Each epoch involves 2000 synaptic weight updates, with our circuit consuming 1–3.24 µW per update compared to 32 µW for conventional CMOS analog multipliers. Over the complete training period, this results in a 76.58% reduction in total power consumption, demonstrating the potential of our approach for energy‐efficient neuromorphic computing. This corresponds to a 76.58% reduction in power consumption, demonstrating the potential of our approach for energy‐efficient neuromorphic computing. The methodology for power measurement and comparative analysis is detailed in Figure S20 (Supporting Information). Comprehensive benchmarking against existing hardware implementations further validates the energy efficiency advantages of our neuromorphic approach across different fabrication technologies (Table S2, Supporting Information). These experimental results confirm the implementation of Hebbian learning in our circuit architecture, providing a pathway for integrating on‐chip learning capability in neuromorphic hardware.

## 3 Conclusion

The integration of STs with TSMs in our ANP represents a significant advancement in hardware‐based neuromorphic computing. Our approach demonstrates that carefully engineered device architectures can enable sophisticated learning mechanisms while maintaining system simplicity. The platform's ability to perform correlation‐based weight updates without requiring extensive peripheral circuitry validates the feasibility of efficient on‐chip learning. Furthermore, the successful demonstration of Hebbian learning principles and signal stability across the array configuration suggests potential for broad applications in neuromorphic computing. While the weight update is physically realized within the device architecture, implementing a fully autonomous feedback loop, addressing the limited driving strength of neuron outputs for multi‐stage connectivity, and enabling closed‐loop, task‐level adaptation remains important future directions for system‐level integration. This work provides critical insights into the practical realization of brain‐inspired computing architectures and establishes design principles for future neuromorphic systems. The demonstrated capabilities in hardware‐based learning and signal processing efficiency indicate promising advancements in energy‐efficient computing platforms.

## 4 Experimental Section

### Device Fabrication—ST Fabrication

The Si/SiO_2_ (100 nm) wafer substrate was cleaned through sequential sonication in acetone, isopropyl alcohol, and deionized water for 20 min each. A 30 nm‐thick ITO layer was deposited on the substrate via radio frequency magnetron sputtering and thermally annealed at 600 °C for 30 min. The ITO layer was patterned using conventional photolithography (AZ 5214E) and subsequently etched with 35 vol% hydrochloric acid diluted in distilled water. Thereafter, a 10 nm IGZO layer was deposited onto the ITO layer via radio frequency magnetron sputtering and thermally annealed at 450 °C for 5 min in the air. Next, the IGZO layer was subjected to conventional photolithography using AZ5214E and then chemically etched with 3 vol% LEC‐12 (Cyantek Co.) diluted in distilled water. An Su‐8 isolation layer was spin‐coated onto the IGZO layer and patterned, and then an Al gate electrode was thermally evaporated through a shadow mask. Afterward, an ion gel ink was prepared by mixing poly(ethylene glycol)diacrylate monomer, 2‐hydroxy‐2‐methlpropiophenone initiator, and [EMIM^+^][TFSI^−^] ionic liquid in a weight ratio of 2:1:21. The prepared ion gel was drop‐cast onto the IGZO layer and patterned under UV radiation (200 mW cm^−2^ at 365 nm for 10 s) using a photomask.

### Device Fabrication—TSM Fabrication

The TSM fabrication process began with the same ITO bottom electrode preparation steps described above. Prior to the active layer deposition, the patterned ITO substrate was subjected to UV‐ozone treatment for 10 min to ensure surface wettability. A Nafion solution was prepared by diluting D‐520 (DuPont Co.) with isopropyl alcohol to achieve a 4 wt.% concentration. This solution was mixed with a UV‐cross‐linkable azide‐based cross‐linker ((oxybis(ethane‐2,1‐diyl))bis(oxy))bis(ethane‐2,1‐diyl) bis(4‐azido‐2,3,5,6‐tetrafluorobenzoate) in a 95:5 wt.% ratio. The prepared Nafion solution was spin‐coated onto the treated ITO substrate at 2000 rpm for 60 s and exposed to UV radiation (1000 W cm^−2^, 254 nm). The device fabrication was completed by thermally evaporating an Ag/Au (40 nm/20 nm) top electrode through a shadow mask.

### Characterization

The electrical characteristics of the STs and TSMs were investigated using a Keithley 4200A‐SCS instrument. The voltage characteristics were measured using Tektronix (MDO3032). All the measurements were conducted under ambient air at 25 °C.

### Hebbian learning

The fundamental principle of Hebbian learning in neural networks is captured by the expression “neurons that fire together wire together,” which can be mathematically formalized as Equation (1):

(1) $$\Delta w=\eta \left(x\cdot y\right)$$

where Δ*w* represents the change in synaptic weight, *η* is the learning rate, x is the presynaptic input, and y is the postsynaptic output. This basic formulation suggests that the connection strength between neurons increases when their activities are correlated. However, this simple form introduces stability issues since continuous positive correlations result in unbounded weight growth. To address this limitation, Oja proposed a modified learning rule that introduces a normalization term *αw·y* ^2^:

(2) $$\Delta w=\eta \left(x\cdot y-\alpha w\cdot y^{2}\right)$$

where α is a normalization factor, and w represents the current weight value. This term serves two crucial functions: First, it prevents unbounded weight growth by introducing a mechanism for weight decay proportional to the square of the postsynaptic activity (*y* ^2^) and the current weight (*w*). Second, it helps maintain the weights within a stable range while preserving the correlation‐learning behavior essential to Hebbian plasticity. This normalization term makes Oja's rule particularly suitable for practical implementations where physical constraints necessitate bounded weight values.

### Computer Simulation

For neural network simulation, a convolutional neural network was implemented using PyTorch. The network architecture comprised a single convolutional layer with 400 kernels (5 × 5), followed by batch normalization, a ReLU activation function, and max pooling (2 × 2). The activation function was implemented as a thresholded linear function *f*(z) ≈ max(0, a(z − b)) to reflect the empirical synaptic update behavior observed in the ST devices, rather than an arbitrarily chosen ReLU activation. The output was then flattened and connected to a fully connected layer with 10 output neurons. The network was trained on the Fashion MNIST dataset using Oja's learning rule with a learning rate of 0.04. Training was performed for 50 epochs with a batch size of 256. This relatively simple architecture was chosen to demonstrate the basic principles of Hebbian learning while maintaining computational efficiency.

## Conflict of Interest

The authors declare no conflict of interest.

## Supporting information



Supporting Information

## Data Availability

The data that support the findings of this study are available from the corresponding author upon reasonable request.
