# Supplementary material for: Hardware Implementation of On‐Chip Hebbian Learning Through Integrated Neuromorphic Architecture
Source: Adv Mater. 2025 Jun 25;37(38):2506920. doi: 10.1002/adma.202506920 (PMC12464636; doi:10.1002/adma.202506920)
Supplement: Supplementary file 1 — Supporting Information [file ADMA-37-2506920-s001.docx]

**Hardware Implementation of On-Chip Hebbian Learning through Integrated Neuromorphic Architecture**

Seonkwon Kim^1,†^, Seongil Im^1,2,†^, In Cheol Kwak^1^, Jungwha Lee^1^, Dong Gue Roe^1^, Hyunsu Ju^2^*, and Jeong Ho Cho^1^*

^1^Department of Chemical and Biomolecular Engineering; Yonsei University, Seoul 03722, Republic of Korea.

^2^Center of Quantum Technology, Korea Institute of Science and Technology (KIST), Seoul 02792, Republic of Korea.

^†^S.K. and S.I. contributed equally to this work

*Correspondence should be addressed to hyunsuju@kist.re.kr (H.J.) and jhcho94@yonsei.ac.kr (J.H.C.).

Keywords: Neuromorphic devices, Neuromorphic computing, Arficial synapse, Artificial neuron, On-chip learning

**Table-of-contents:**

1. **Figure S1:** Effect of the Ga content in IGZO on threshold voltage modulation.
2. **Figure S2:** Potentiation behavior of the ST under low-amplitude and low-frequency pulse inputs.
3. **Figure S3:** PPF characteristics of the STs.
4. **Figure S4:** Heatmap depicting the spatial distribution of the LTP characteristics across the 6 × 6 synaptic transistor array.
5. **Figure S5:** Individual LTP characteristics of all STs in the 6 × 6 array.
6. **Figure S6:** *V*_G_-dependent synaptic characteristics of the IGZO-based STs.
7. **Figure S7:** Frequency- and pulse width-dependent synaptic characteristics of the IGZO-based STs.
8. **Figure S8:** Bias stress stability test of the IGZO-based ST.
9. **Figure S9:** Operational stability test of the IGZO-based ST under repeated LTP/D cycles
10. **Figure S10:** Dual-gate IGZO-based ST.
11. **Figure S11:** Transfer characteristics of dual-gate STs.
12. **Figure S12:** Effect of inter-pulse interval on the response of the ST under varying gate voltages.
13. **Figure S13**: Individual I-V characteristics of all TSMs in the 6×6 array.
14. **Figure S14:** Extracted threshold voltages of all 36 TSM devices over 30 cycles each.
15. **Figure S15:** Electrical characteristics of Nafion-based TSM with different film thicknesses.
16. **Figure S16:** Effects of circuit parameters on neuron characteristics.
17. **Figure S17:** Spatial distribution of input patterns and the corresponding array response.
18. **Figure S18:** Individual *V*_feed_ characteristics across the 6 × 6 array.
19. **Figure S19:** Multi-input signal integration capability of the artificial neural platform.
20. **Figure S20:** Power consumption comparison between the feedback-ST circuit and a conventional CMOS analog multiplier.
21. **Table S1:** Two-stage training protocol, network architecture, and quantitative benchmark
22. **Table S2:** Energy per weight-update for representative on-chip learning engines


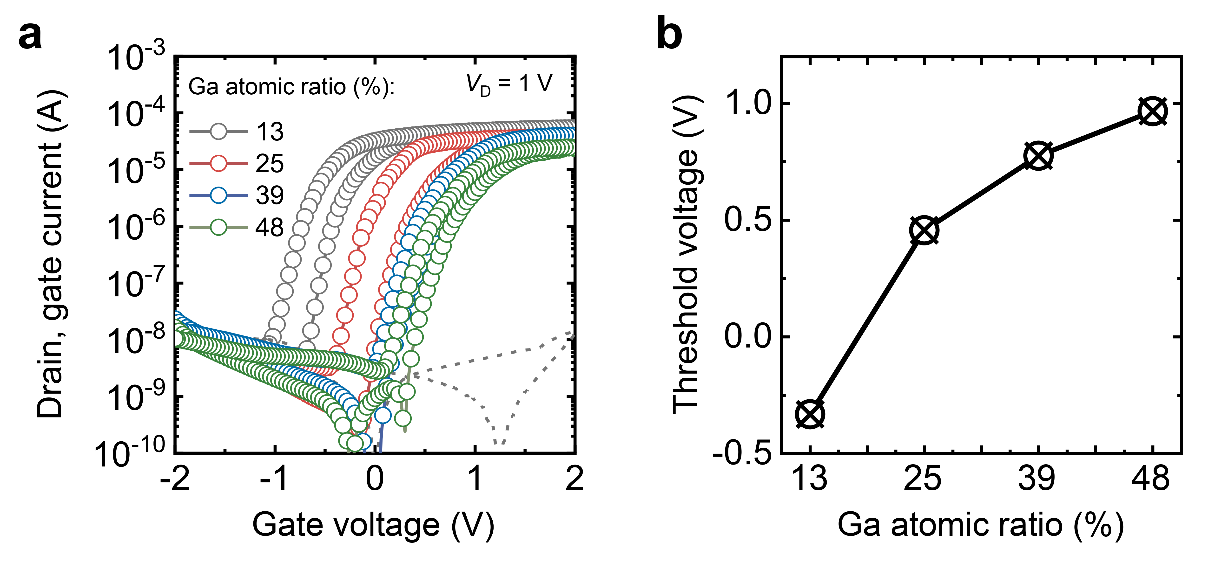


**Figure S1. Effect of the Ga content in IGZO on threshold voltage modulation. a**) Transfer curves of devices with varying Ga atomic ratios. **b**) Threshold voltage as a function of Ga atomic ratio. The threshold voltage increased from −0.33 V to 0.97 V as the Ga content in IGZO increased from 13% to 48%. High Ga contents in IGZO suppress oxygen vacancy formation, thereby reducing electron doping since oxygen vacancies act as shallow electron donors.

*
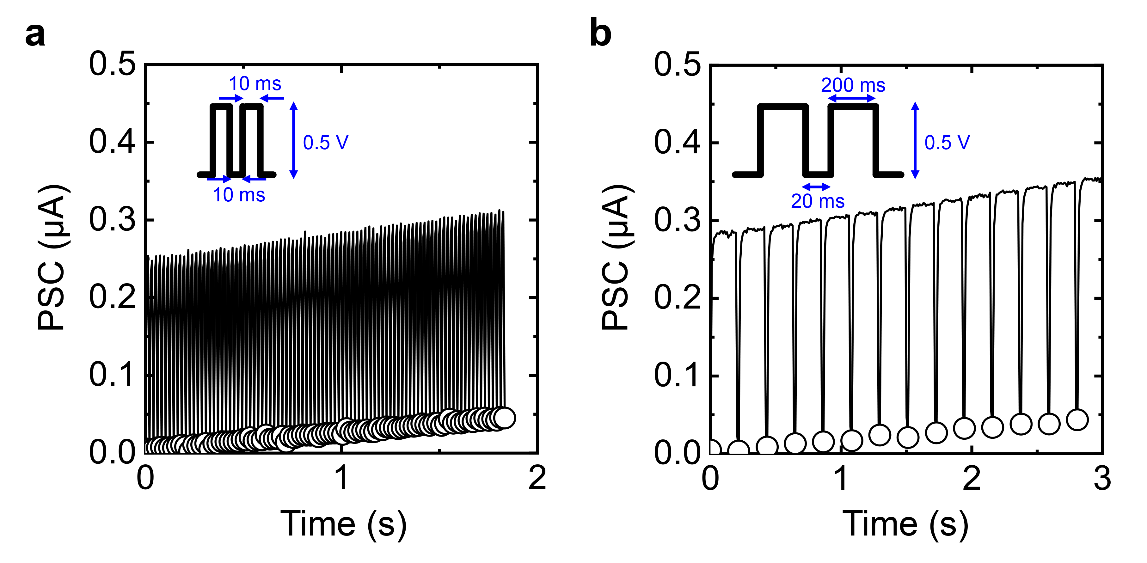
*

**Figure S2. Potentiation behavior of the ST under low-amplitude and low-frequency pulse inputs. a**) Pulse train of 0.5 V amplitude at 50 Hz. **b**) Pulse train of 0.5 V amplitude with sub-10 Hz frequency and extended pulse widths (200 ms).

The ST was tested under input conditions that resemble the output characteristics of the neuron device presented in this work. When pulse trains with sub-10 Hz frequency and 200 ms pulse width were applied—mimicking the spike characteristics of our neuron device—the ST again exhibited effective and accumulative potentiation. These results confirm that the feedback-ST can operate reliably under voltage and timing conditions representative of our system-level implementation.


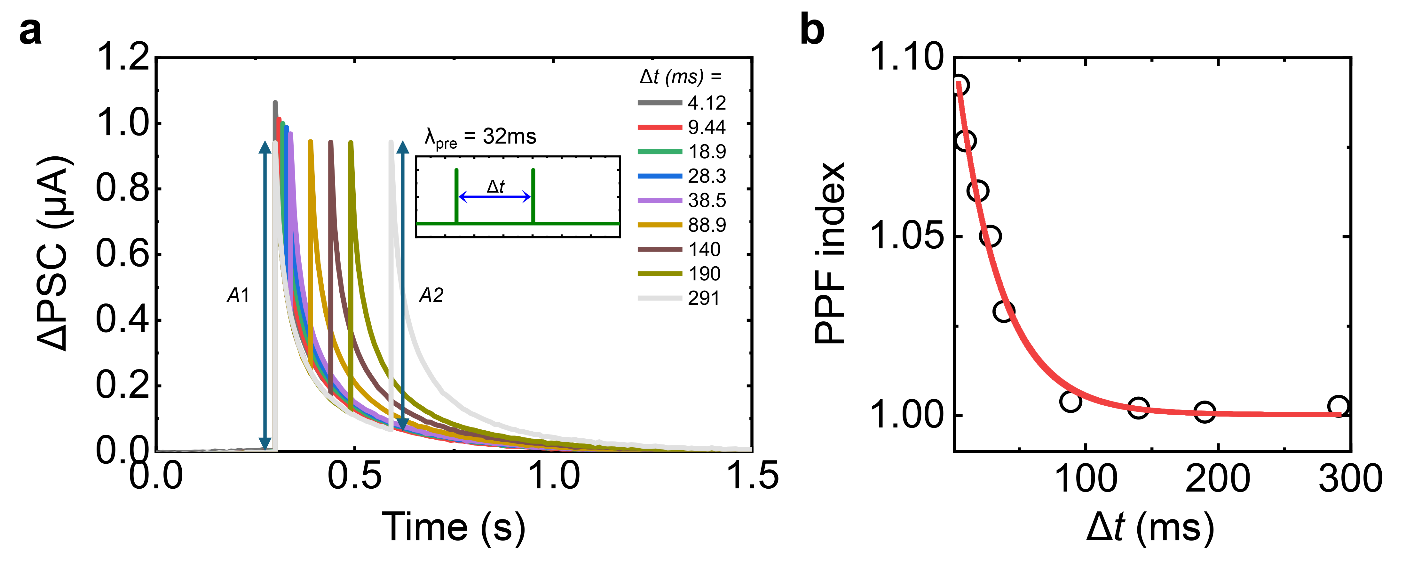


**Figure S3. PPF characteristics of the STs. a**) PPF response when two consecutive pulses are applied with varying time intervals (Δt). **b**) PPF index (*A*2/*A*1) as a function of pulse interval Δt.

**
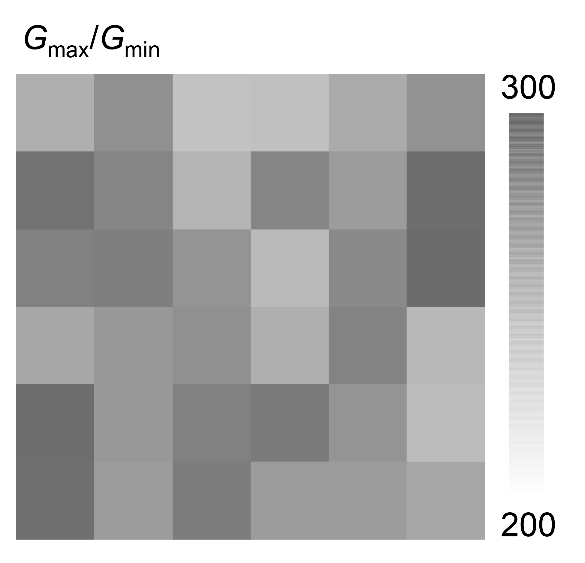
**

**Figure S4. Heatmap depicting the spatial distribution of the LTP characteristics across the 6 × 6 synaptic transistor array.**


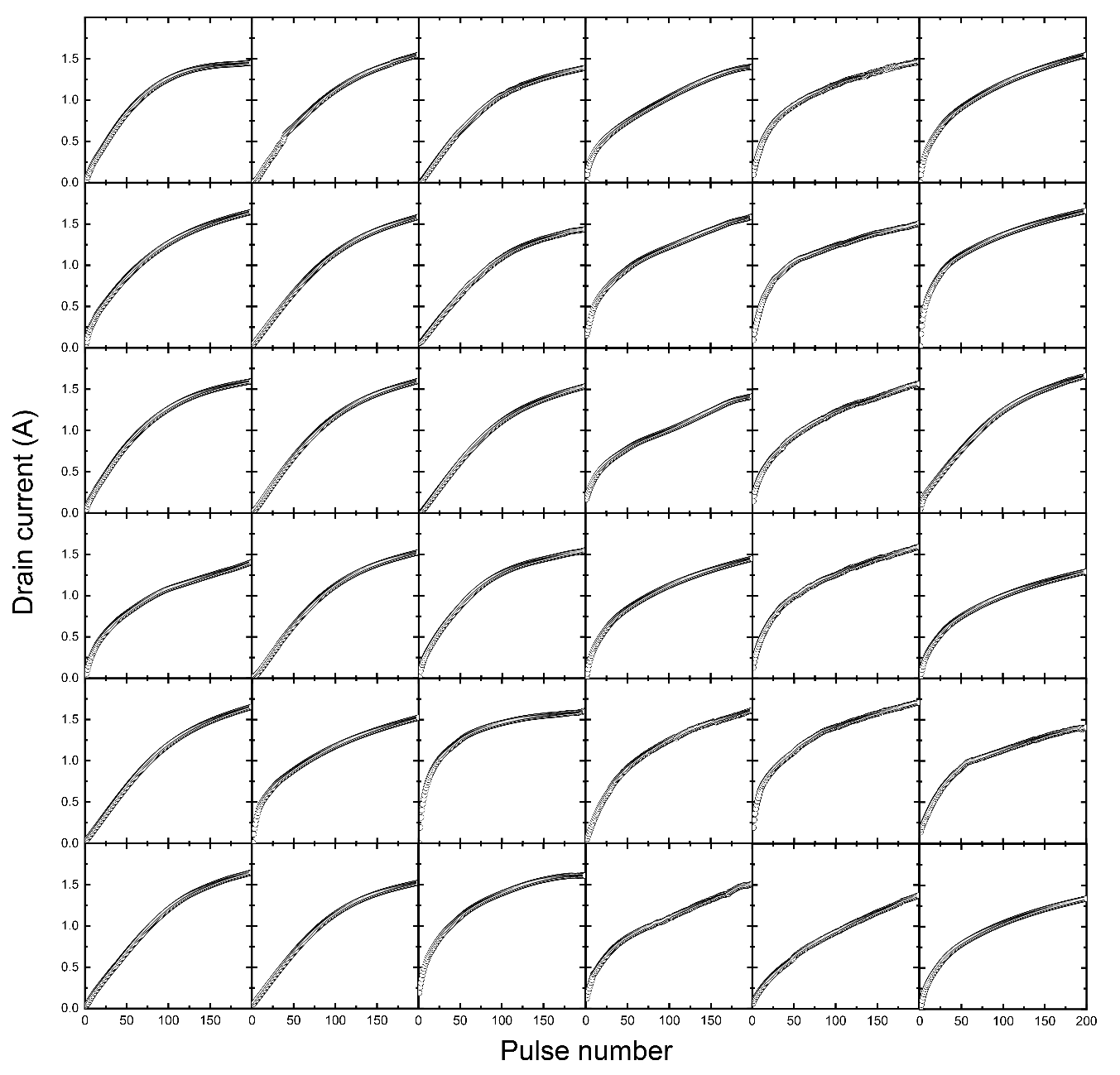


**Figure S5. Individual LTP characteristics of all STs in the 6 × 6 array.** The measurements were conducted with *V*_G_ = 2 V, pulse width = 131 ms, and pulse frequency = 7.45 Hz.


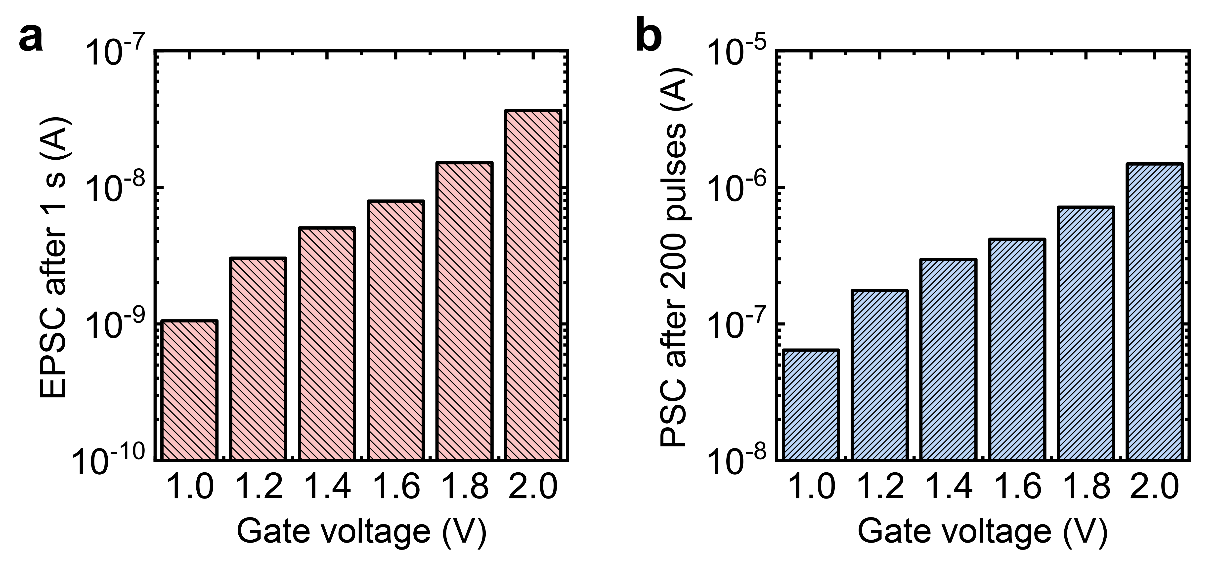


**Figure S6. *V*_G_-dependent synaptic characteristics of the IGZO-based STs. a**) EPSC measured at 1 s for different *V*_G_s, showing an increase from 1.04 × 10^−9^ A at *V*_G_ = 1.0 V to 3.65 × 10^−8^ A at *V*_G_ = 2.0 V. **b**) PSC measured after 200 consecutive pulses, demonstrating an increase from 6.43 × 10^−8^ A at *V*_G_ = 1.0 V to 1.49 × 10^−6^ A at *V*_G_ = 2.0 V.


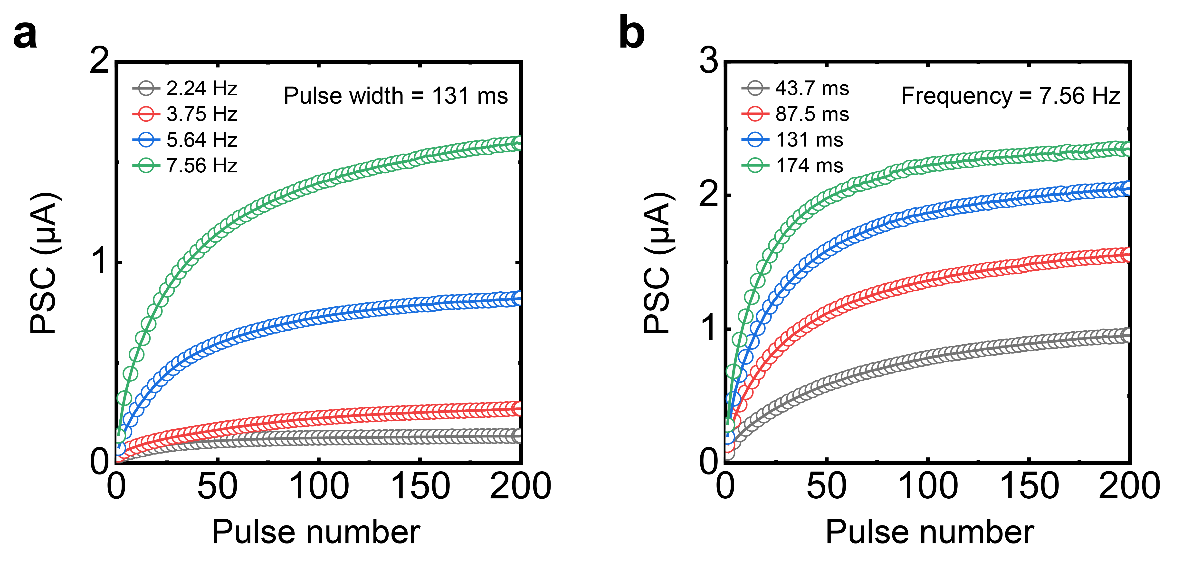


**Figure S7. Frequency- and pulse width-dependent synaptic characteristics of the IGZO-based STs. a**) PSC measured under varying pulse frequencies at a fixed pulse width (131 ms). **b**) PSC measured under varying pulse widths at a fixed frequency (7.56 Hz).

***
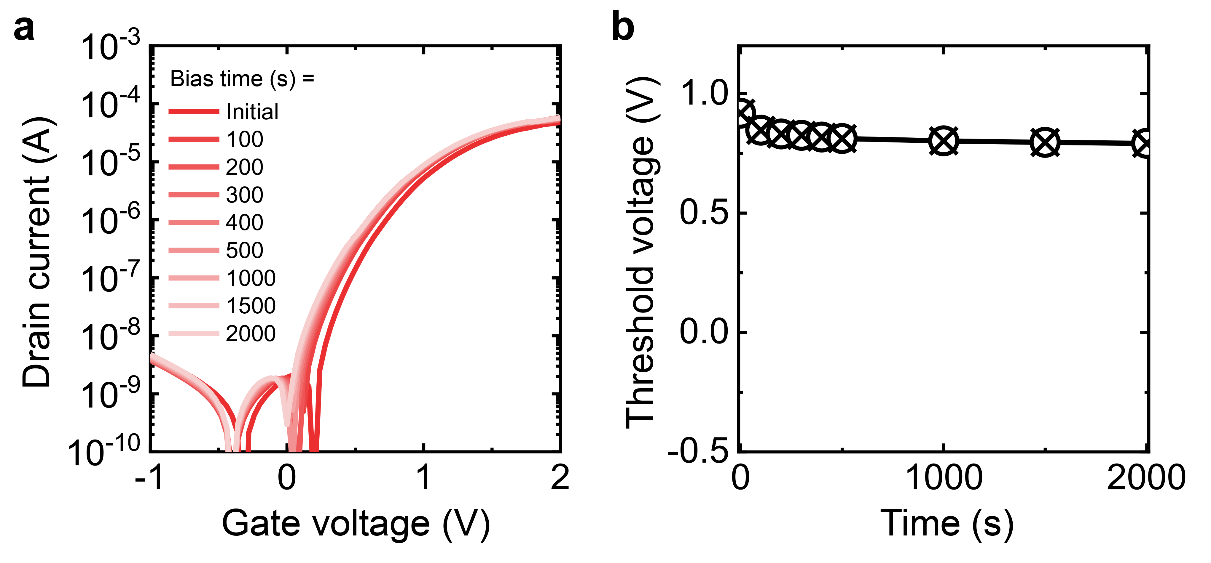
***

**Figure S8. Bias stress stability test of the IGZO-based ST. a)** Transfer curves measured during a bias stress test in which a constant gate voltage of -0.5 V was applied for up to 2000 seconds. **b**) Extracted threshold voltage as a function of stress time, showing a minimal shift from 0.918 V to 0.792 V (Δ*V*_TH_ = 0.126 V).

***
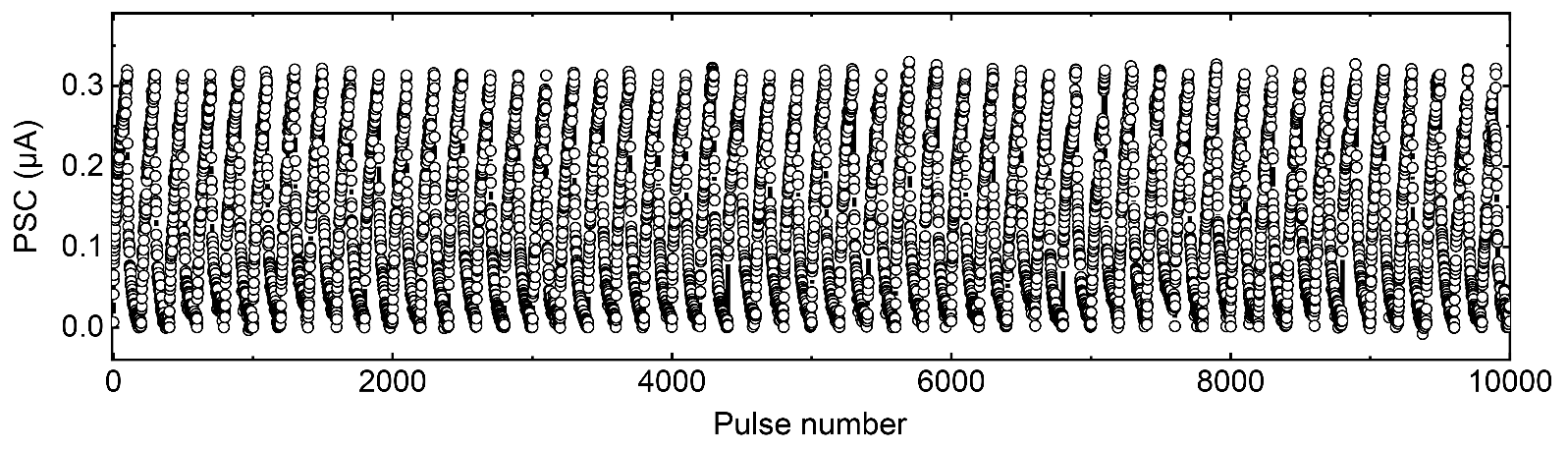
***

**Figure S9. Operational stability test of the IGZO-based ST under repeated LTP/D cycles (*V*_LTP_ = 1.4 V, *V*_LTD_ = -0.5 V).**

***
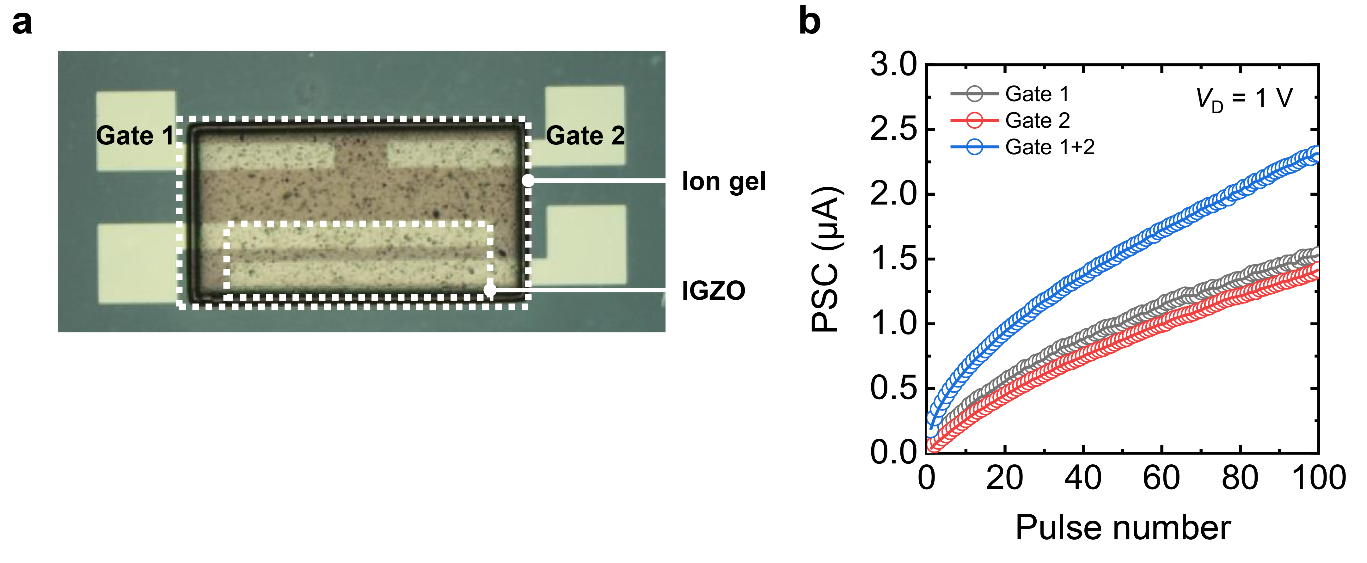
***

**Figure S10. Dual-gate IGZO-based ST. a**) Device structure of the dual-gate ST. **b**) Pulse response characteristics of the dual-gate ST under various gate biasing conditions.

**
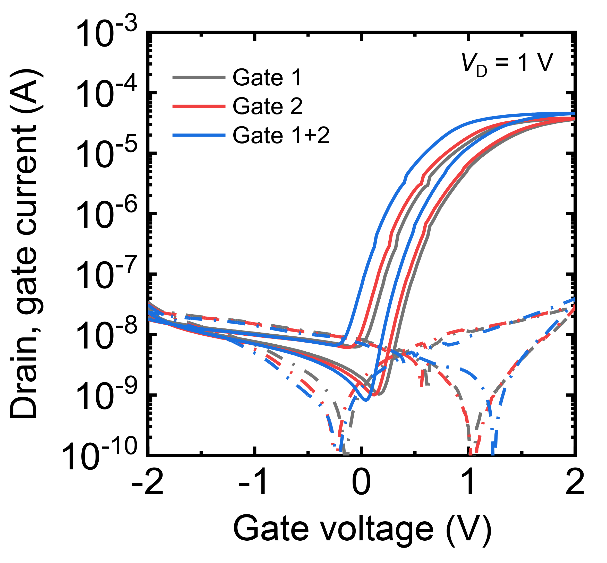
**

**Figure S11. Transfer characteristics of dual-gate STs.** Transfer characteristics of dual-gate STs exhibiting independent sweeps of gate 1 (with gate 2 grounded) and gate 2 (with gate 1 grounded) and simultaneous sweeps of both gates, demonstrating the individual and combined effects of dual-gate operation.

***
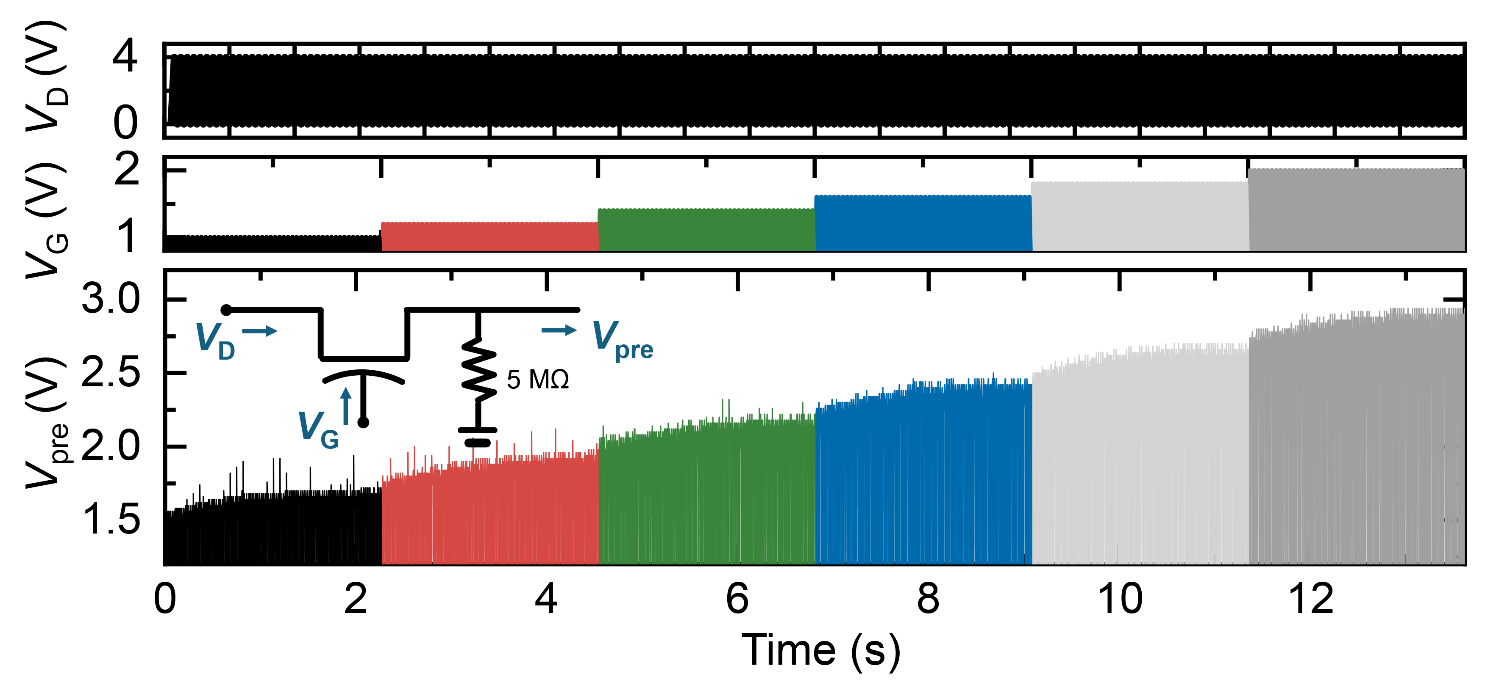
***

**Figure S12. Effect of inter-pulse interval on the response of the synaptic transistor (ST) under varying gate voltages.**

**
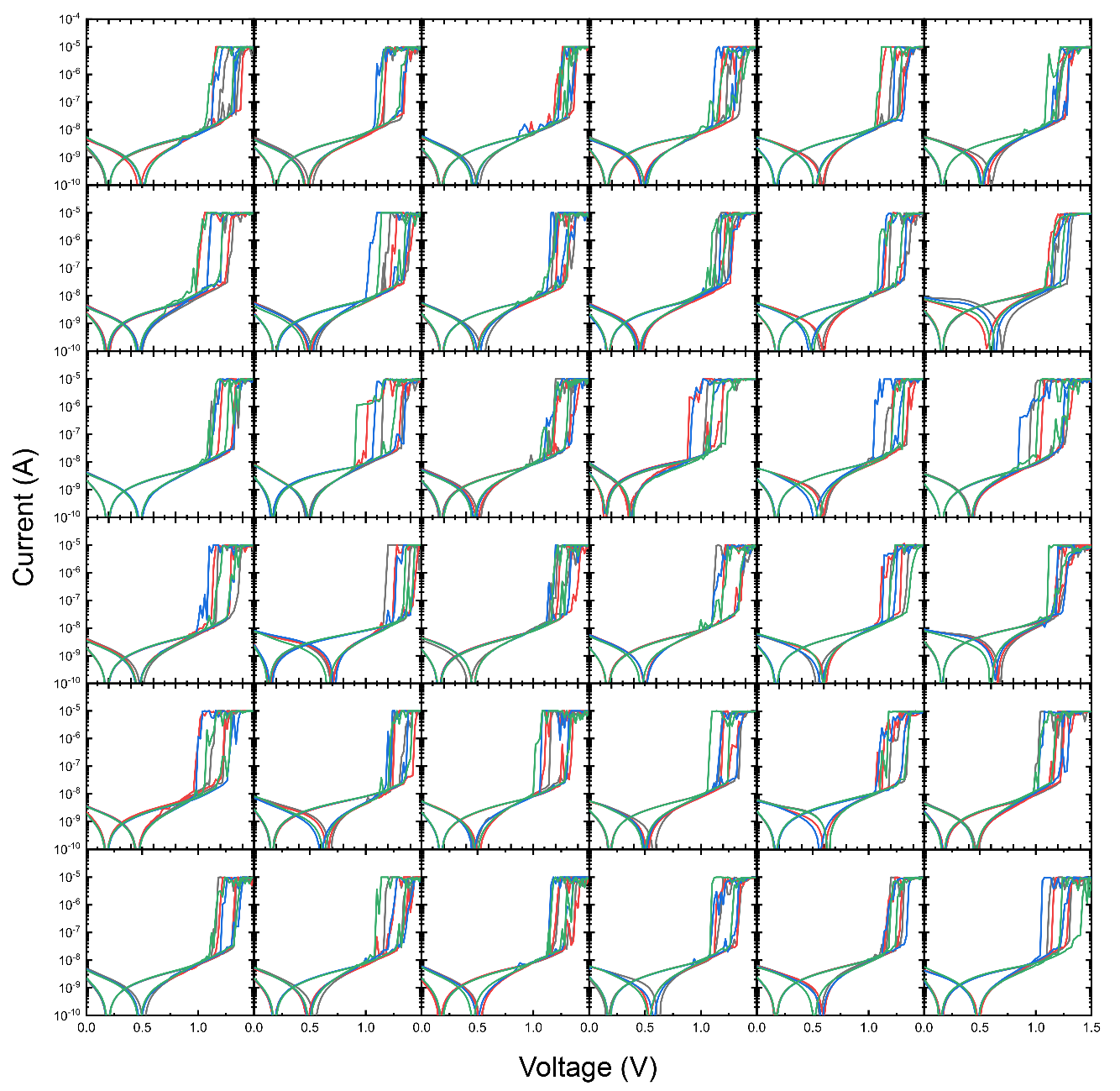
**

**Figure S13. Individual I-V characteristics of all TSMs in the 6×6 array. I-V curves collected from all 36 TSM devices over 30 switching cycles each.**

**
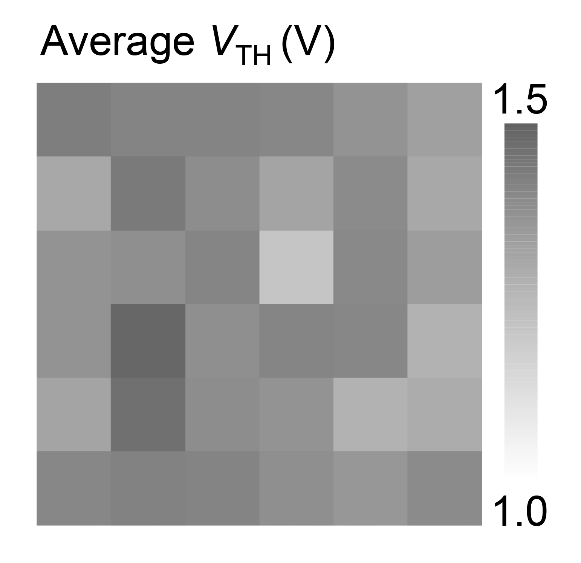
**

**Figure S14. Extracted threshold voltages of all 36 TSM devices over 30 cycles each.**

**
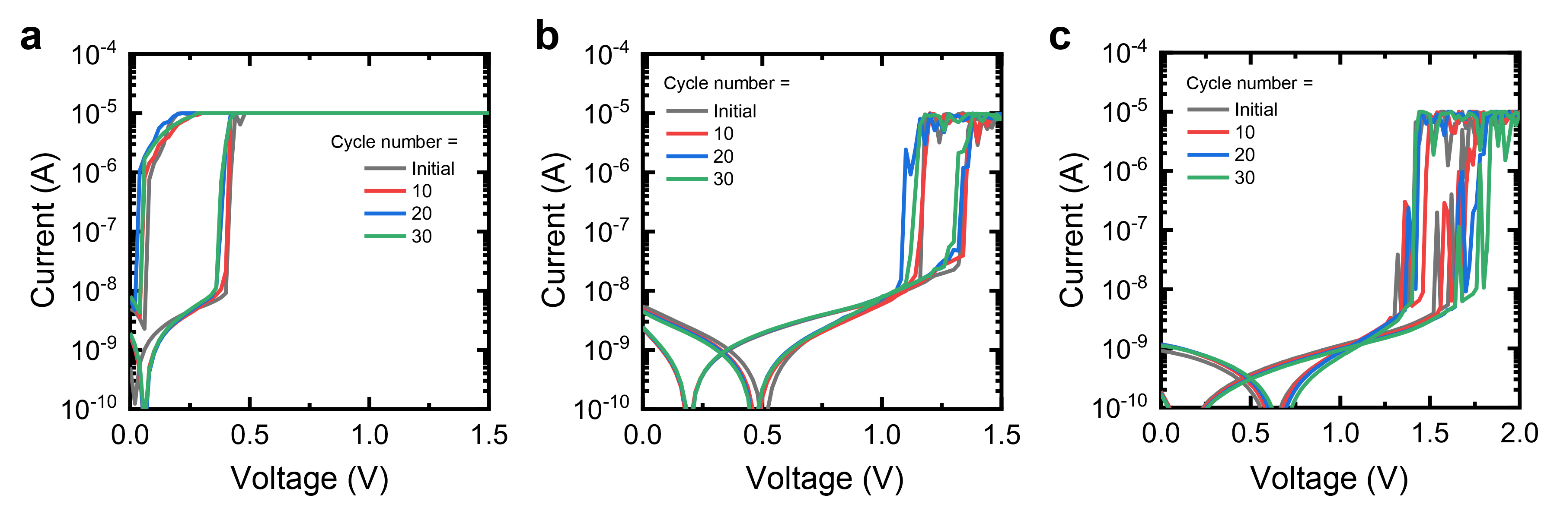
**

**Figure S15. Electrical characteristics of Nafion-based TSM with different film thicknesses. a)** 103.5 nm, **b)** 171.6 nm, **c)** 223.0 nm**.** As the Nafion thickness increases, the threshold voltage shifts to higher values. This trend is attributed to delayed formation of Ag conductive filament in thicker films, which likely results from longer ion transport paths within the ionic matrix.

**
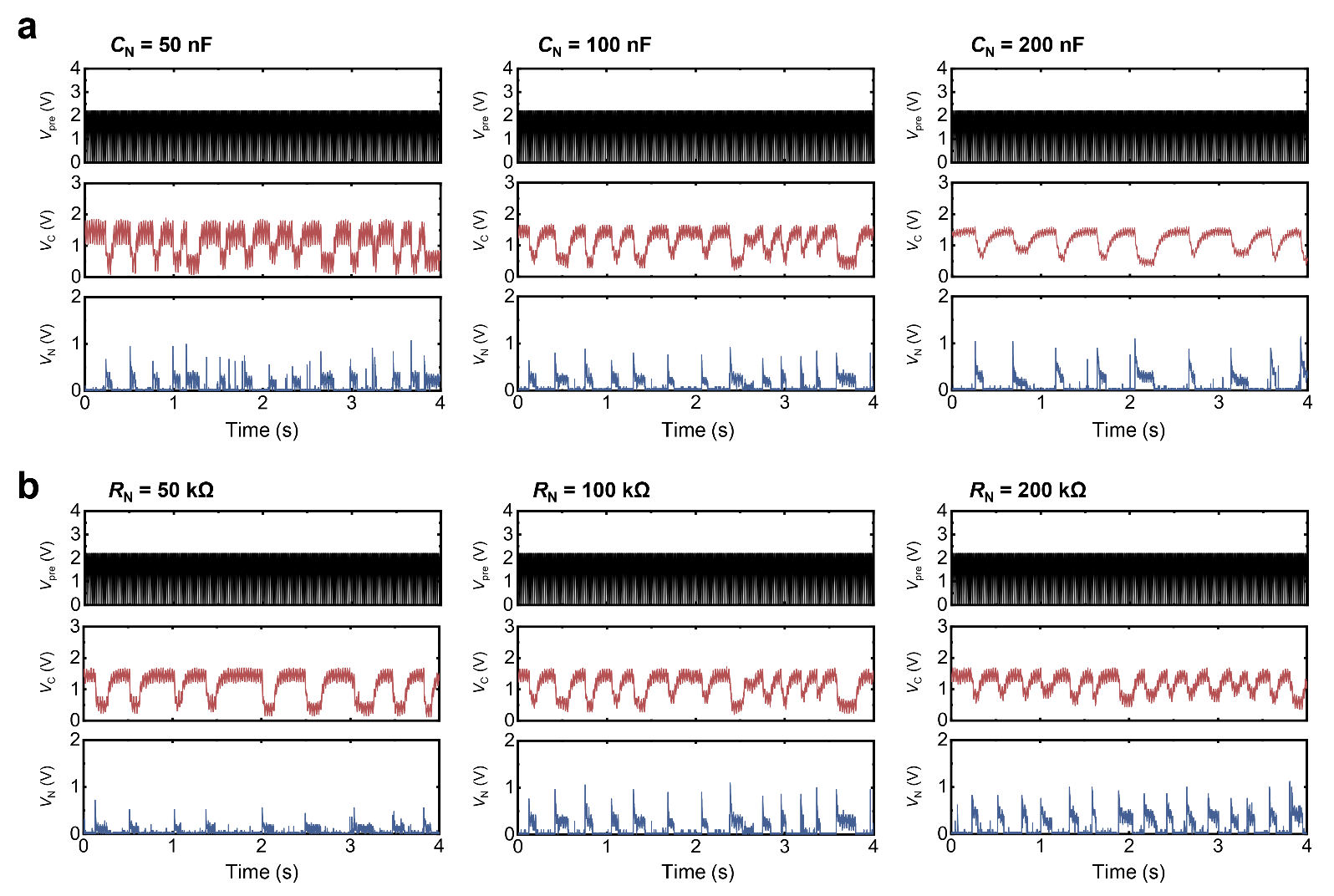
**

**Figure S16. Effects of circuit parameters on neuron characteristics. a**) Measurement of capacitor voltage (*V*_C_) and neuron output (*V*_N_) under different capacitances (*C*_N_ = 50 nF, 100 nF, and 200 nF). Increased *C*_N_ results in decreased firing frequencies because of the increased charging time constant. **b**) Impact of discharge loop resistance (*R*_N_ = 50 kΩ, 100 kΩ, and 200 kΩ) on neuron behavior. A large *R*_N_ increases the amplitude through voltage division, but it decreases firing frequency because of an increased RC time constant in the discharge loop.

The observed behavior can be explained by considering the relationship between circuit parameters and TSM dynamics. In the charging phase, the time constant *τ*_C_ = *R*_s_*C*_N_ determines how quickly the capacitor voltage reaches the threshold voltage. Large *C*_N_ values require additional time to reach *V*_TH_, resulting in low firing frequencies, which allow for precise control of the temporal response of the neuron. During the discharge loop, the time constant *τ*_D_ = *R*_N_*C*_N_ determines the capacitor discharge rate through the TSM in its LRS. Increasing the *R*_N_ enhances the output amplitude through voltage division but simultaneously retards the discharge process. The slower discharge leads to incomplete capacitor reset, which shortens the subsequent integration time allows the capacitor to reach the *V*_TH_ more quickly in the next cycle—thus increasing the firing frequency. This trade-off between output amplitude and frequency demonstrates how circuit parameters can be tuned to achieve desired neuron characteristics.

**
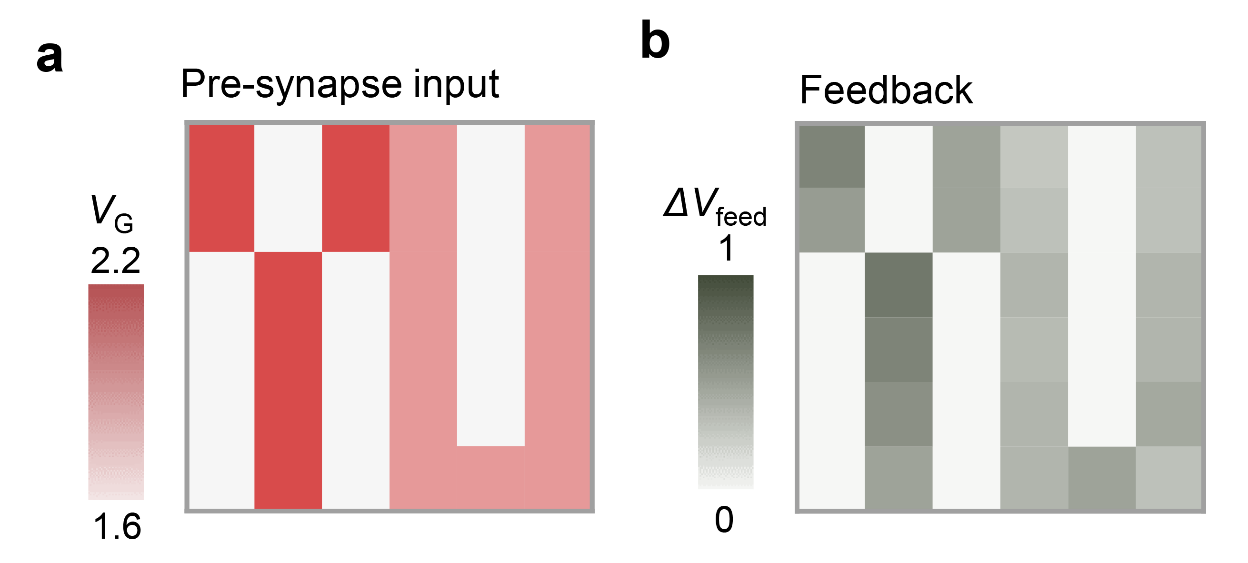
**

**Figure S17. Spatial distribution of input patterns and the corresponding array response. a**) Input voltage pattern consisting of a Y-shaped region with 2.0 V (left) and a U-shaped region with 1.8 V (right) applied to the 6 × 6 array. **b**) Heatmap showing the spatial distribution of Δ*V*_feed_ (defined as the change in *V*_feed_ before and after *V*_G_ application) across the array. The response demonstrates voltage-dependent potentiation: a negligible change where *V*_G_ = 0 V, moderate potentiation in regions with 1.8 V input, and strong potentiation where 2.0 V was applied, reflecting the analog signal processing capability of the array.

**
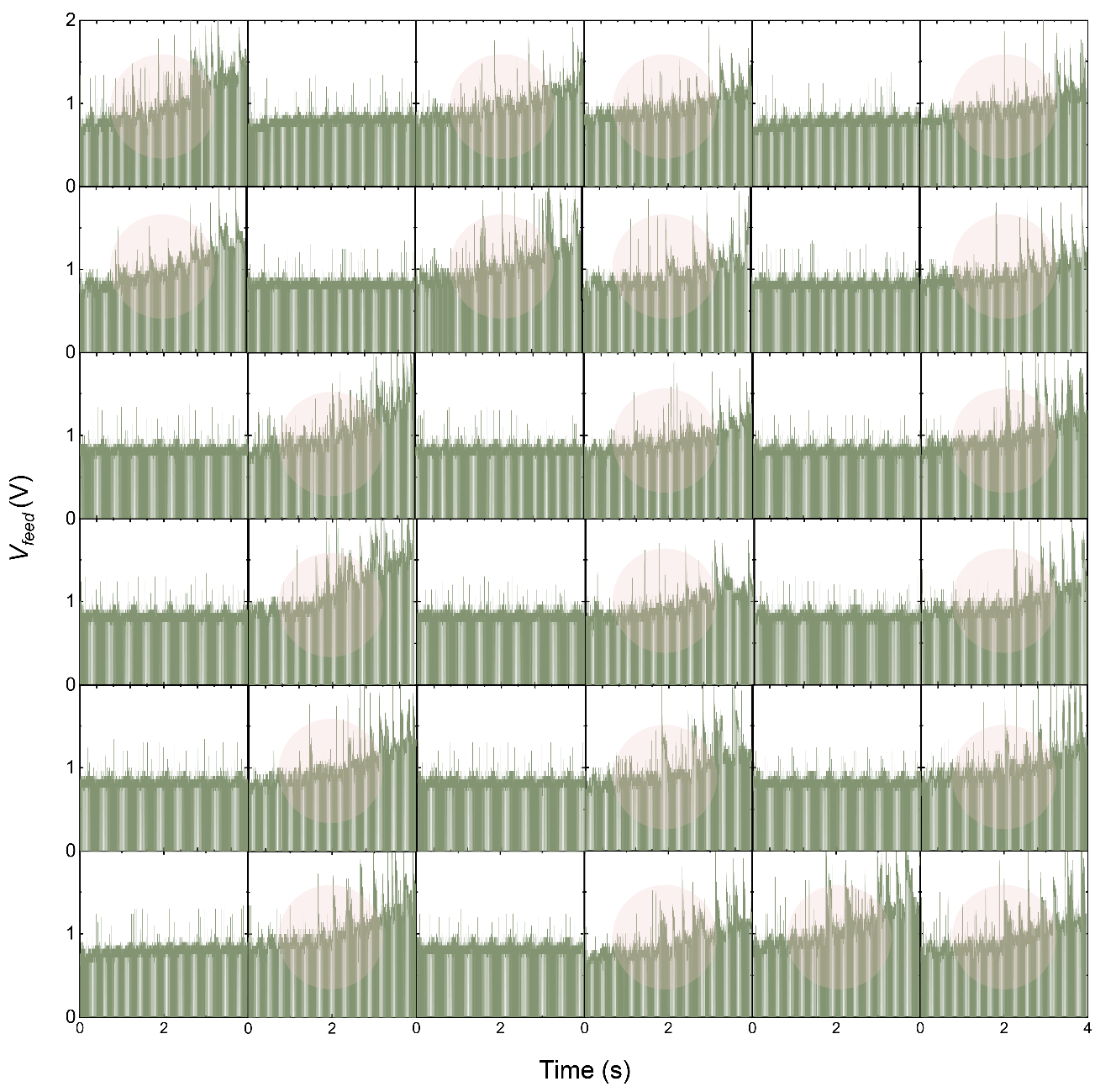
**

**Figure S18. Individual *V*_feed_ characteristics across the 6 × 6 array.** *V*_feed_ measurements from all 36 devices in the array, demonstrating device-to-device variations and the overall response uniformity of the array under the applied voltage patterns.

*
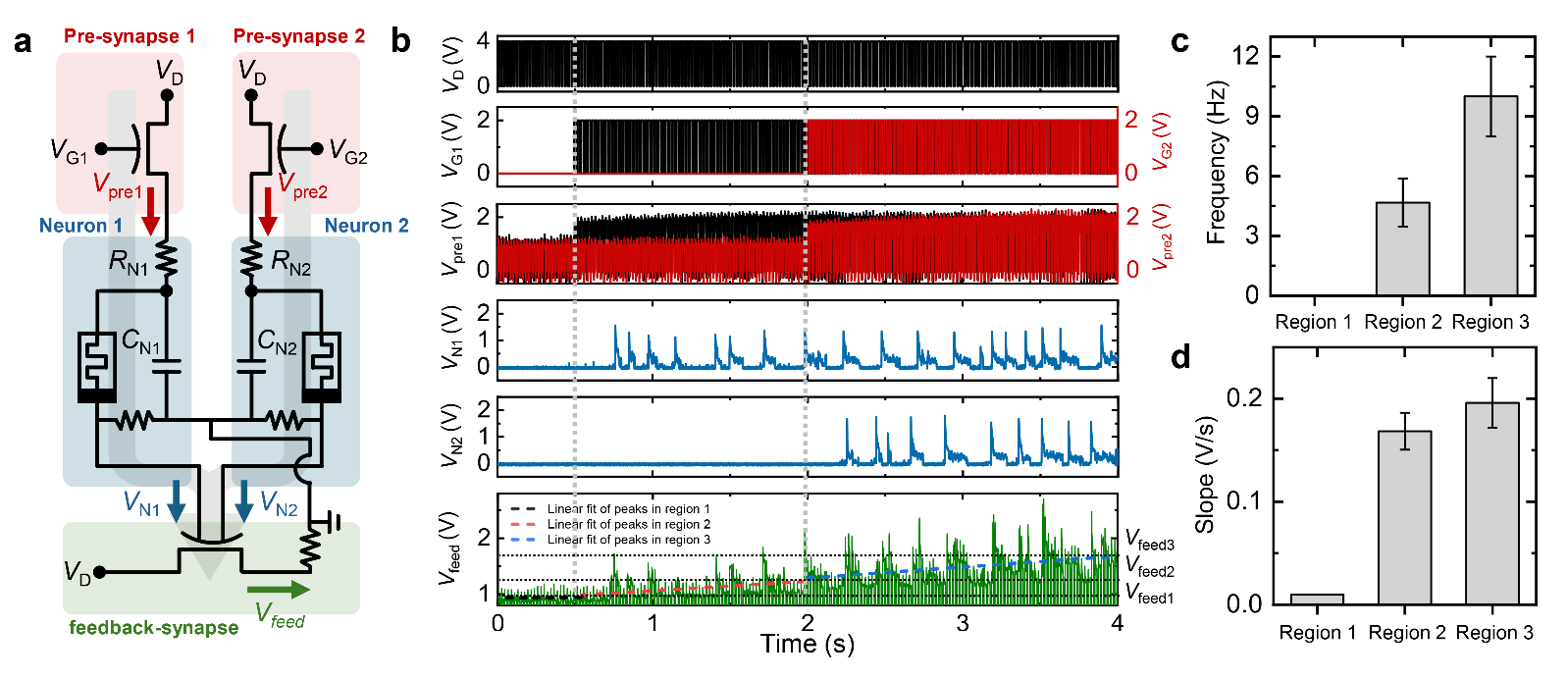
*

**Figure S19. Multi-input signal integration capability of the artificial neural platform. a**) Schematic illustration of two neuron outputs combined at a single feedback synapse. **b**) Signal transmission characteristics across three distinct operating regions: region 1 (no input at *V*_G1_ and *V*_G2_), region 2 (only *V*_G1_ applied), and region 3 (both *V*_G1_ and *V*_G2_ applied). **c**) Combined neuron output frequency (*V*_N1_ + *V*_N2_) in each region, demonstrating frequency doubling in region 3 compared to region 2. **d**) *V*_feed_ potentiation slopes demonstrating enhanced signal integration: a negligible slope in region 1, 0.168 V s^−1^ in region 2, and 0.196 V s^−1^ in region 3 with dual inputs.

**
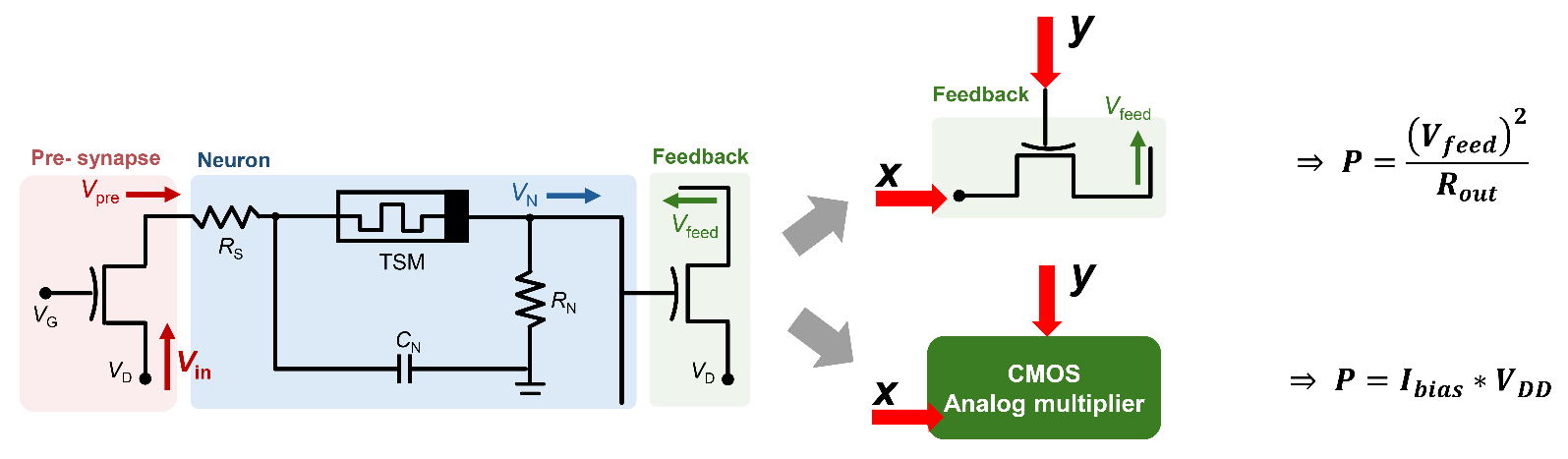
**

**Figure S20. Power consumption comparison between the feedback-ST circuit and a conventional CMOS analog multiplier.** The fundamental computation in Hebbian learning involves the multiplication of presynaptic and postsynaptic signals (*x*·*y*), which are represented as continuous analog values. To achieve this, our circuit utilizes the feedback voltage (*V*_feed_) as a function of the input (*x*) and output (*y*) signal. In our feedback-ST circuit, power dissipation during a synaptic update(Δ*w*) is determined by the feedback voltage (Δ*V*_feed_) and the output resistance (*R*_out_). For the worst scenario, *R*_out_ is set to the minimum 1 MΩ for the maximum power consumption, leading to a power dissipation range of 1 μW (at *V*_feed_ = 1 V) to 3.24 μW (at *V*_feed_ = 1.8 V) per synaptic update. These calculations are derived from voltage transformations that directly map to weight updates (Δ*w*), as implemented in our computational model. For comparison, we consider a conventional CMOS analog multiplier, which is commonly designed using the widely adopted Gilbert Cell architecture for analog signal processing. The power consumption in such multipliers is primarily dictated by the bias current (*I*_bias_) and the supply voltage (*V*_DD_), which together define the total static and dynamic power dissipation. A well-optimized implementation operates at an estimated power consumption of 32 μW per multiplication operation. This estimation is based on known low-power analog multiplier designs, where *I*_bias_ typically falls in the range of tens to hundreds of μA, and *V*_DD_ operates within a standard low-power supply range (e.g., 1 V to 1.2 V). Additionally, we conducted simulations to convert Δ*w* values into voltage levels and calculated the corresponding power dissipation per synaptic update (2000 updates per epoch).

**Table S1. Two-stage training protocol, network architecture, and quantitative benchmark.**

| **Stage** | **Layer** | **Output dimension** | **In CNN** | **Ours** |
| --- | --- | --- | --- | --- |
| **Backbone** | Conv 5×5, 400 kernel | 400 × 14 × 14 | Back-propagated | Hebbian learning |
|  | Flatten | 78400 | Back-propagated | Hebbian learning |
| **Classifier** | Linear | 10 | Back-propagated | Back-propagated |
| **Test Accuracy** |  |  | **91.2%** | **91.6%** |

The convolutional backbone is first trained for 100 epochs by unsupervised Hebbian learning on the Fashion-MNIST data set and then frozen. A linear classification head that represents only ~2 % of the total parameters is subsequently trained for 20 supervised epochs with the available labels. For a direct, like-for-like benchmark an identically configured network is trained end-to-end by conventional back-propagation for the same overall budget of 120 epochs. Despite using no labels during the computationally intensive feature-extraction phase, the Hebbian-pre-trained model achieves a higher test accuracy (91.6 %) than the fully supervised baseline (91.2 %), demonstrating that local unsupervised learning can match—and marginally exceed—conventional performance while offering the practical benefit of label-free feature formation. The table lists the exact layer composition, parameter counts, and final test accuracies for both training strategies.

**Table S2. Energy per weight-update for representative on-chip learning engines.**

| **Work** | **Process / V_DD_** | **Energy**  **/MAC[µW]** | **ADCs/DACs**  **+write-ICs[µW]** | **Energy**  **/update[µW]** |
| --- | --- | --- | --- | --- |
| **This work (Hebbian)** | 180 nm / 1.8 V | – | – | 1 – 3 |
| **SRAM IMC + SGD^[1]^** | 28 nm / 0.9 V | 2 | 3 | 5 |
| **1T1R crossbar + BP^[2]^** | – / 1 V | 5 | 10 | 15 |
| **CMOS digital MAC^[3]^** | 65 nm / 1.1 V | 32 | ≥ 70 | > 100 |

Unlike backpropagation implementations requiring substantial ADC/DAC circuitry for analog signal processing, our Hebbian synapse achieves weight updates through simple charge integration, eliminating data converter requirements. Individual Hebbian updates require only single local multiplication (x·y), whereas backpropagation necessitates multiple MAC operations (forward pass, backward pass, gradient computation) plus verification and write cycles. These architectural advantages result in significant energy efficiency improvements across different fabrication processes and circuit designs, demonstrating the practical viability of our neuromorphic approach.

**References**

[1] Su, Jian-Wei, et al. "15.2 A 28nm 64Kb inference-training two-way transpose multibit 6T SRAM compute-in-memory macro for AI edge chips." 2020 IEEE International Solid-State Circuits Conference-(ISSCC). IEEE, 2020.
[2] Baba, H., et al. "Novel analog in-memory compute with> 1 nA current/cell and 143.9 TOPS/W enabled by monolithic normally-off Zn-rich CAAC-IGZO FET-on-Si CMOS technology." 2021 IEEE International Electron Devices Meeting (IEDM). IEEE, 2021.
[3] Yang, Jiyue, et al. "A 65nm 8-bit all-digital stochastic-compute-in-memory deep learning processor." 2022 IEEE Asian Solid-State Circuits Conference (A-SSCC). IEEE, 2022.
